# The role of cell membrane-coated nanoparticles as a novel treatment approach in glioblastoma

**DOI:** 10.3389/fmolb.2022.1083645

**Published:** 2023-01-04

**Authors:** Pantea Allami, Arash Heidari, Nima Rezaei

**Affiliations:** ^1^ Network of Immunity in Infection, Malignancy and Autoimmunity (NIIMA), Universal Scientific Education and Research Network (USERN), Tehran, Iran; ^2^ School of Medicine, Tehran University of Medical Sciences, Tehran, Iran; ^3^ Research Center for Immunodeficiencies, Children’s Medical Center, Tehran University of Medical Sciences, Tehran, Iran; ^4^ Students’ Scientific Research Center, Tehran University of Medical Sciences, Tehran, Iran; ^5^ Department of Immunology, School of Medicine, Tehran University of Medical Sciences, Tehran, Iran

**Keywords:** glioblastoma multiform, cell membrane, nanoparticles, coating, nanotechnology, treatment

## Abstract

Glioblastoma multiform (GBM) is the most prevalent and deadliest primary brain malignancy in adults, whose median survival rate does not exceed 15 months after diagnosis. The conventional treatment of GBM, including maximal safe surgery followed by chemotherapy and radiotherapy, usually cannot lead to notable improvements in the disease prognosis and the tumor always recurs. Many GBM characteristics make its treatment challenging. The most important ones are the impermeability of the blood-brain barrier (BBB), preventing chemotherapeutic drugs from reaching in adequate amounts to the tumor site, intratumoral heterogeneity, and roles of glioblastoma stem cells (GSCs). To overcome these barriers, the recently-developed drug-carrying approach using nanoparticles (NPs) may play a significant role. NPs are tiny particles, usually less than 100 nm showing various diagnostic and therapeutic medical applications. In this regard, cell membrane (CM)-coated NPs demonstrated several promising effects in GBM in pre-clinical studies. They benefit from fewer adverse effects due to their specific targeting of tumor cells, biocompatibility because of their CM surfaces, prolonged half-life, easy penetrating of the BBB, and escaping from the immune reaction, making them an attractive option for GBM treatment. To date, CM-coated NPs have been applied to enhance the effectiveness of major therapeutic approaches in GBM treatment, including chemotherapy, immunotherapy, gene therapy, and photo-based therapies. Despite the promising results in pre-clinical studies regarding the effectiveness of CM-coated NPs in GBM, significant barriers like high expenses, complex preparation processes, and unknown long-term effects still hinder its mass production for the clinic. In this regard, the current study aims to provide an overview of different characteristics of CM-coated NPs and comprehensively investigate their application as a novel treatment approach in GBM.

## 1 Introduction

Glioma is a type of Central Nervous System (CNS) tumor originating from glial cells. Based on the World Health Organization’s (WHO) classification, gliomas are divided into four grades. Grade I is curable with surgery. Grade II is not much proliferous but has a high potential to recur. Grade III is more invasive and proliferous than the first two grades. However, the most malignant, invasive, and deadliest type of glioma is grade IV, known as glioblastoma multiform (GBM) (Rajaratnam et al., 2020). GBM is the most malignant glioma and accounts for more than 60% of primary brain tumors in adults (Janjua et al., 2021). To date, there has not been any effective treatment for GBM. The median survival after diagnosis is between 12.5–18 months. Moreover, the 5-year survival rate of GBM patients is less than 5% (Hsu et al., 2021; Mathew et al., 2022).

Patients with GBM may experience various symptoms based on their tumor location. Symptoms usually start with severe headaches. They may involve mood disorders, dementia, weakness, and unconsciousness (Alexander and Cloughesy, 2017). Magnetic resonance imaging (MRI) is the gold standard imaging method for diagnosing GBM. Unfortunately, GBM can only be diagnosed with MRI or its suggestive symptoms when the tumor has developed to an advanced stage, making the treatment process much harder (Yang et al., 2022). The standard treatment of GBM is resection surgery combined with radiotherapy and chemotherapy. These therapies may increase the survival rate, but they have not been successful as a permanent cure, as they cannot prohibit GBM recurrence. Thus, it is clear that a revolution in GBM therapy strategies is needed (Tan et al., 2020a).

Numerous factors make GBM treatment challenging, but the three most important are glioblastoma stem cells (GSCs), tumor heterogeneity, and the blood-brain barrier (BBB). In this regard, GSCs are responsible for tumor heterogeneity, GBM radio-resistance, chemo-resistance, and recurrence. GSCs are not entirely definable. They may originate from neural stem cells or transformed astrocytes. They are heterogeneous and do not represent similar biomarkers, though some biomarkers, like the cluster of differentiation (CD)133, CD15, CD44, integrin-α, and A2B5, are common among them. The first important step toward effective treatment of GBM is the well-recognition of GSCs and finding a way to target and restrain them, which is shown to be complicated and impossible with a single therapy modality (Nakod et al., 2018; Gimple et al., 2019; Hsu et al., 2021).

On the other hand, BBB is composed of endothelial cells with tight junctions and adhesion molecules that forms a barrier between blood and brain extracellular fluid. BBB serves as an obstacle to many small and large molecules, including drugs and other therapeutic agents (Patel and Patel, 2017; Attia et al., 2021). To better cross BBB, either BBB must get more permeable, or the approach to drug delivery must be modified (Hsu et al., 2021). To enhance BBB permeability, mechanical and chemical potentials can be used. A targeted ultrasounds wave is an example of a mechanical approach; ultrasound can temporarily open BBB with minor damage in specific areas for 24 h (Zhang et al., 2022). Vascular endothelial growth factor (VEGF) injection can also increase BBB permeability in small doses. However, GBM cells secrete VEGF naturally, and this molecule is responsible for angiogenesis in the tumor site. Therefore, although studies claim that injected VEGF effects on BBB are transient, it is not an ideal approach as it can lead to neurogenesis and tumor progression (Wen et al., 2017; Lundy et al., 2018; Cha et al., 2020). Another approach for drug delivery to the brain is convection-enhanced delivery, which can provide different types of drugs in various sizes directly to the tumor’s local area using an implantable catheter (Ung et al., 2015). However, this method has physical limitations like backflow, air bubbles, white matter edema, and targeted heterogeneity (Mehta et al., 2017).

Nanomedicine can introduce a novel drug delivery system, which has the potential to penetrate BBB and specifically target GSCs. Nanoparticles (NPs) are tiny materials ranging from 1–100 nm (nm) (Jadoun et al., 2022). They can be classified into different groups based on their size, morphology, and chemical and physical properties. They usually have complex compositions and comprise three main layers: surface, shell, and core. The core is the most important layer, and one of the classifications of NPs is based on their core materials. NPs possess different characteristics making them suitable options for carrying drugs and therapeutic agents to the CNS and tumor sites. They stay in the blood circulation for an extended period and allow the release of the carried drugs as per the intended dose, enabling them to have fewer serum fluctuations and adverse effects (De Villiers et al., 2008). NPs also penetrate the targeted organs easily, thanks to their tiny sizes. They can also assist drug uptake by the targeted cells and allow efficient drug delivery (Patra et al., 2018). The other properties of NPs include their stability and biocompatibility (Gavas et al., 2021). There are some organic/polymeric NPs, like dendrimers, micelles, or liposomes, and some inorganic, like metal-based and silica-based NPs. NPs can get loaded with various therapeutic agents (Shergalis et al., 2018). Moreover, nano-carrier surfaces can get modified for advanced targeted drug delivery systems. The targeted drug delivery agents can be drug carrier encapsulated cells (Attia et al., 2021) or conjugated-biomimetic agents. NPs less than 100 nm can pass BBB and reach the tumor site due to the enhanced permeability retention (EPR) effect. Modifying ligand-protein agents on the NPs surface, which can bind to endothelial cells’ receptors in BBB, could make transportation through BBB faster and easier (Thangudu et al., 2020; Chen et al., 2022). Biomimetic agents provide targeted delivery and more prolonged drug circulation in the body, which used to be applied with synthetic materials like polyethylene glycol (PEG). The most advanced approach in biomimetics is cell-membrane (CM) coating technology (Rampado et al., 2022). Therefore, regarding GBM malignancy and the urgency to find the cure, and since there have been progressive studies about CM-coated NPs for GBM, this review aims to investigate the recent advances in CM-coated NPs as a novel technology to overcome significant barriers to GBM treatment.

## 2 An overview on NPs in GBM treatment

### 2.1 Insight into nanotechnology in tumor treatment and nano-carrier classifications

Nanotechnology has become a pioneer in industry, agriculture, and medicine (Rasool et al., 2022). Besides their natural therapeutic qualities, they are carriers for many agents for various medical applications like drug delivery and treatment, imaging and diagnosis, and even prevention and vaccination (Shi et al., 2017). NP carriers provide several benefits, including safe and long drug delivery. Using pharmaceutical drugs in some therapeutic methods, like chemotherapy, as a primary cancer treatment might have serious side effects. However, targeted drug delivery with the help of nano-carriers reduces the detrimental effects of these drugs (Fang et al., 2018; Gawel et al., 2022). For drug delivery, nano-carriers could reach the tumor sites both passively and actively. In the passive route, extensive blood vessels caused by tumor-induced angiogenesis and defects in lymphatic drainage could extend the release and sustain of nano drugs in the tumor site. On the other hand, active targeting uses bio-mimetic agents like CM vesicles for more effective delivery (Liu et al., 2022a). The mechanism by which CM-coated NPs could penetrate BBB varied according to the NP type and carried drug and, as mentioned, can be generally classified into passive and active routes (Bhaskar et al., 2010). The passive route is an energy-independent process, facilitating the simple diffusion of small lipophilic particles through BBB’s endothelial cells. For instance, gold NPs can use this mechanism thanks to the EPR effect induced by brain tumors like GBM (Zhou et al., 2018). On the other hand, active transporting routes are energy-dependent and require adenosine three phosphates hydrolysis to produce the required energy source. They can be divided into specific receptor-mediated or unspecific adsorption-mediated endocytosis and also carrier-mediated transport. Receptor-mediated endocytosis is triggered by the specific interconnection between NPs and cerebral endothelial receptors and, as an example, can be applied by transferrin-conjugated NPs (Grabrucker et al., 2016; Zhou et al., 2018). Moreover, adsorption-mediated endocytosis is based on electrostatic interactions between the positively charged molecules and negatively-charged surfaces of cerebral endothelial cells. Cationic NPs, like gold NPs can reportedly utilize this mechanism to cross BBB (Zhou et al., 2018). In gliomas, BBB hinders therapeutic molecules’ entrance. Also, passive targeted drug delivery is not possible in GBM treatment. Hence, bio-mimetic-based nano-carriers, which can provide a long-sustained and fast release of therapeutic agents in tumor sites with a low dosage of drug consumption, could be breakthroughs in GBM treatment (Mathew et al., 2022).

Nano-carriers and NPs are varied in size, surface charge, shape, and type. Each of these qualities plays a specific role in penetrating BBB and cellular uptake; therefore, they should be considered in designing a drug delivery approach (de Almeida et al., 2021; Sargazi et al., 2022). The size of the NP is between 5–500 nm, with an optimal size of less than 200 nm (Hsu et al., 2021). It must be considered that NPs must be cloaked since they have hyper-reactive surfaces and are destructive to the body’s tissues and organs (Megarajan et al., 2022). The surface charge of NPs is also critical because it determines NPs' behavior in biological environments. In this regard, the positive charge of NPs proved to be more suitable for drug delivery (de Almeida et al., 2021). Furthermore, based on the targeted cells, different shapes of NPs could have different amounts of cellular uptake (de Almeida et al., 2021). On the other hand, nano-carriers vary based on their preparation method. There are three types of nano-carriers, including nanocapsules, nanospheres, and NPs. NPs are the most critical nano-carriers in GBM treatment (Hsu et al., 2021), which are categorized into different types, including metal-based NPs (Gold, magnetic, and quantum dots), silica-based NPs, and synthetic-polymeric NP (Fang et al., 2018; Hsu et al., 2021; Gawel et al., 2022) Figure 1.

**FIGURE 1:**
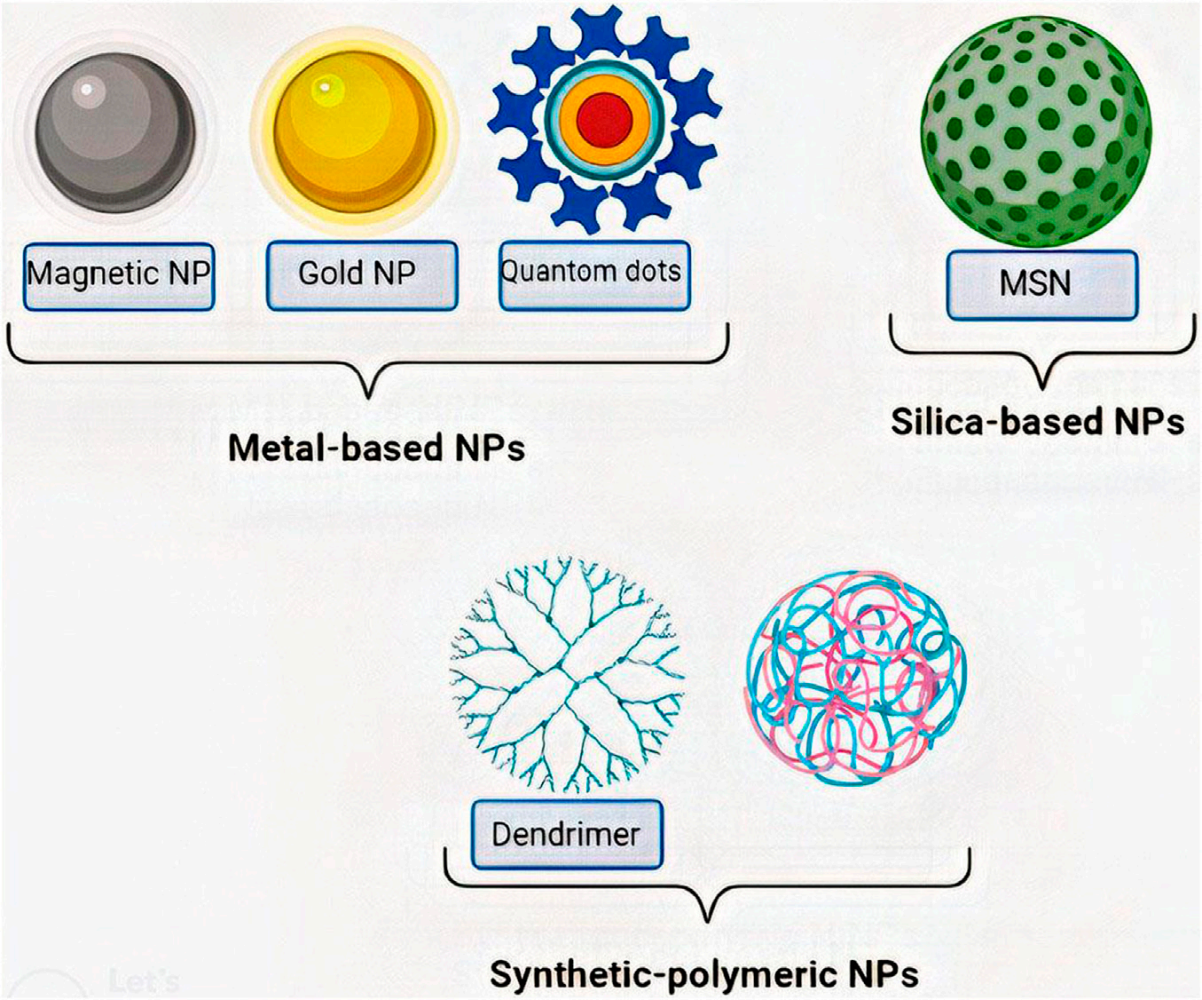
Classes of most important NPs. NPs are classified into various types, among which metal-based NPs (Gold, magnetic, and quantum dots), silica-based NPs (MSN), and synthetic-polymeric NP (Dendrimer and PLGA) are the most important ones. MSN:Mesoporous silica nanoparticle, PLGA: Polylactic-co-glycolic acid.

CM-coated NPs pose several advantages compared to multiple targeted NPs. As CM-coated NP surfaces imitate CM functionality, they can reportedly diminish the body’s immune responses to the NPs. Moreover, CM-coated NPs benefit from fewer adverse effects, acceptable toxicity, improved biocompatibility, and prolonged half-life compared with synthetic NP-based drug carriers (Luk et al., 2016; Yaman et al., 2020a; Han et al., 2021). For instance, the resulting complex from coating CM with drug-loaded NPs can diminish the clearance by the reticuloendothelial system, and thereby, CM-NPs could sustain in blood circulation for a more extended period (Han et al., 2021). Moreover, CM decreases the toxicity of the NPs and therefore enhances the biocompatibility of the CM-NP complexes leading to fewer adverse effects on other systems, as drug-loaded NPs cannot be released until they reach their targeted organs (Han et al., 2021). The other primacy of CM-NPs is that they can be easily equipped with targeting ligands using chemical reactions or physical interconnections (Han et al., 2021). Also, their surface can be engineered to exert the desired immunomodulatory effects on their targeted sites, which can be used for tumor regression (Yaman et al., 2020a). Hence, the superiorities of CM-NPs over the targeted modified NPs can be summarized in their multifunctional abilities resulting from CM properties, allowing them to escape the immune system to target tumor sites specifically (Gao et al., 2013; Yaman et al., 2020a).

Three mechanisms are suggested as probable causes of NP-induced cell intoxication. The first is the interaction between NP surfaces and CM, inducing apoptotic signaling cascades. The second is the interconnection between NP and surrounded interstitial tissue of the targeted cells, leading to depletion or upregulation of different ions and proteins, which are fundamental for cells. The third mechanism is the internalization of the NPs into the cells, resulting in the activation of several downregulating signals leading to cell death. In the third mechanism, which is the most accepted theory for NP-induced cell intoxication, NPs are internalized through the cells by endocytosis (Safi et al., 2011). Afterward, they degrade in the lysosomes and produce calcium and phosphor ions. The high calcium concentration resulting from the fast internalization of NPs could lead to cell death. This mechanism was shown in a study by (Bonany et al., 2022). They targeted MG-63 cells with two NPs, namely hydroxyapatite (HA) NPs and magnesium-doped HA NPs. The authors observed with the calcium-fluorescent probes that a high number of calcium-rich vesicles are found after introducing NPs into cell culture. Moreover, they revealed with cryo-soft X-ray imaging that several microvascular bodies in the targeted cells exist, which play roles as calcium stores (Bonany et al., 2022). In this regard, these two approaches could be utilized to assess each NP’s cell toxicity.

### 2.2 NPs classification and their approach in GBM treatment

As mentioned, there are various types of NPs, which can carry therapeutic agents besides the natural therapeutic effects of some of them. This section introduces different classes of NPs and their history in GBM treatment (Table 1).

**TABLE 1:** A summary of important NPs in GBM and their essential characteristics.

Nanoparticle		Characteristics
Metal-based	Gold	Biological stable and biocompatible with simple synthesis and low toxicity- immune tolerant- Modifiable surface Liu and Peng (2017); Ajdary et al. (2018)appropriate for PTT and radiotherapy Jenkins (2015); Gawel et al. (2022)-FDA-approved NP Lee et al. (2022)
	Magnetic	Intercellular hyperthermia-induced agents Gawel et al. (2022)- MRI imaging agents
		Biocompatible-get magnetically guided to the tumor site Tapeinos et al. (2019a)
		Essential examples: SPIONs and Mno_2_
	Quantum-dots	Size: between 2–10 nm
		Semicinductor crystal material
		Intrinsic cytotoxin
		Appropriate for PTT
		Biocompatible Perini et al. (2020a); Gawel et al. (2022)
		Chemotherapeutic carriers with anti tumoral features Perini et al. (2020a); Perini et al. (2020b)
		Essential examples: goldwith antituand graphene quantum dots Wahab et al. (2019)
Silica-based		Examples: MSN is an excellent biocompatible and comprehensive utilization in biomedical Tang et al. (2012); Luo et al. (2019).
		Various mixtures of SiO_2_ with other NPs for an increase in stability Wu et al. (2021).
Polymeric		Examples: PLGA, with the most used in GBM treatment and drug delivery Kapoor et al. (2015), and PLA, with the oldest history in medical approaches Kapoor et al. (2015); Sun et al. (2015); Zhang et al. (2021), PEI appropriate for DNA delivery Han et al. (2021), dendrimers appropriate for RNA delivery Stenström et al. (2018)

PTT, Photothermal therapy; FDA, Food and drug administration; NP, Nanoparticle; SPIONS, Superparamagnetic iron oxide nanoparticles; Mno_2_, Manganese dioxide; MSN, Mesoporous silica nanoparticle; SiO_2_, Silicon dioxide; PLGA, Polylactic-co-glycolic acid; GBM, Glioblastoma multiform; PLA, Poly lactic acid; PEI, polyethylene imine.

#### 2.2.1 Metal-based NPs

Metal-based NPs have several properties, making them appropriate in nano-medicine. The medical application of metal NPs in diseases like cancer, depression, and infections is well-known. They can bind to biological molecules and initiate particular cell signals (He et al., 2021; Rajadurai et al., 2021). For instance, it has been reported that MgFe-layered double hydroxide NPs could efficiently replace the leukemia inhibitory factor in maintaining self-renewing and pluripotency features of mice’s embryonic stem cells by initiating several downregulating signals. Therefore, they could serve as an affordable substance for these cells’ cultivation (He et al., 2021). Another example of the properties of silver-based NPs in exerting antidepressant, anxiolytic and antitumoral effects was reported in a mice study (Rajadurai et al., 2021). Besides, metal-based NPs are resistant to different environments and are also biocompatible, which is crucial when used in the body. Their sensitivity to light also makes them great candidates for photo-based cancer therapies (Habibullah et al., 2021). Several studies confirm the ability of specific metallic NPs to pass through BBB. NPs like gold or silver can cause a pro-inflammatory response in brain microvessel endothelial cells and increase their permeability (Liu et al., 2017). Metal-based NPs are also radiosensitizers and can increase the sensitivity of tumor cells to radiotherapy (Liu et al., 2018). Such properties have inspired scientists to design several therapeutic approaches based on metal NPs (Gawel et al., 2022). The effect of many metal NPs like gold NPs, magnetic NPs, and quantum dots NPs has been measured in GBM pre-clinical and clinical studies. It should be noted that each nanoparticle has its unique features and characteristics, causing each of them to exert its specific effect despite binding to a single molecule. In this regard, a study showed that gold or silver NPs stabilized with the same molecule demonstrated different catalytic and efficacy features. For instance, gold NPs were reported to be better catalysts than silver NPs’ although they showed poor antimicrobial activity compared to silver NPs (Megarajan et al., 2022).

##### 2.2.1.1 Gold NPs

Gold NPs are one of the most popular metal-based NPs in biomedicine and drug delivery, with outstanding features like flexibility, biological stability, simple synthesis, and controllable toxicity (Liu and Peng, 2017; Ajdary et al., 2018). Furthermore, as gold NPs do not trigger immune reactions and are primarily toxic-free, they could easily reach and accumulate in the tumor sites (Singh et al., 2018). Gold NPs, as carriers for chemotherapeutic agents, have American food and drug administration (FDA) approval (Lee et al., 2022). They have been used in GBM treatment as photosensitizer agents in photothermal therapy (PTT) and radiotherapy in pre-clinical studies. In phase zero of a clinical study, gold NPs were used as a carrier for RNA interference (RNAi) (Jenkins, 2015; Gawel et al., 2022). They have a modifiable surface, making them a convenient choice for active targeting drug delivery approaches. In this context, (Peng et al.,2020) modified gold NPs’ surfaces with aptamers to enhance their antitumor efficiency and targeted drug delivery in GBM treatment. In another study, (Wang et al.,2021) modified gold NPs with antibodies against the ephrin type-A receptor for targeted delivery of Temozolomide (TMZ) to the tumor site. There is no reported study of gold NPs coated with CM for GBM treatment, although (Sun et al.,2020) coated gold NPs with cancer CMs for PTT and radiotherapy in oral squamous cancer.

##### 2.2.1.2 Magnetic NPs

Magnetic NPs are great candidates for combined therapies in GBM. Besides their quality as nano-carriers, they can induce hyperthermia in cells after exposure to an alternating magnetic field (AMF) (Gawel et al., 2022). Intracellular hyperthermia has several impacts, including overexpression of the heat shock protein family, production of reactive oxygen species (ROS), and an increase in the fluidity of the cytoplasm membrane. All of these factors could trigger apoptosis in cancer cells (Del Sol-Fernández et al., 2019). Hyperthermia is usually associated with chemotherapy in GBM treatment when using magnetic NPs. For instance, hyperthermia-inducing magnetic NPs like Iron oxide could carry chemotherapeutic agents like TMZ and doxorubicin (DOX) (Carvalho et al., 2019; Afzalipour et al., 2021). The term superparamagnetic iron oxide nanoparticles (SPIONs) is referred to iron oxide magnetic NPs that can carry drugs to a specific tumor site using targeted delivery approaches. SPIONs can be magnetically guided to the tumor site, can be used in MRI, and can kill cancer cells by inducing hyperthermia and ROS production (Tapeinos et al., 2019a). SPIONs’ targeted delivery is possible with different biomaterials. These biomaterials can be conjugated with ligands like small chain or cycle peptides (Siow et al., 2018; Del Sol-Fernández et al., 2019), lipid-based receptors (Tapeinos et al., 2019b), folic acid (Afzalipour et al., 2021), and hyper-branched phenylboronic acid (Song et al., 2019), or they can be a whole CM like a cancer CM (Tapeinos et al., 2019a; Wang et al., 2022).

Besides SPIONs, another magnetic NP that can create ROS and initiate the apoptosis pathway is manganese dioxide (MnO_2_). MnO_2_ does so by scavenging H_2_O_2_. The process also produces O2 inhibiting hypoxia-inducible factor-1α (HIF-1α). HIF-1α induces vascularization and enhances GSC renewal (Gong et al., 2020; Raza et al., 2021; Rasool et al., 2022). In this regard, (Tapeinos et al., 2019a) used MnO_2_ and Fe_2_O_3_ with chemotherapeutic drugs coated with cancer CMs for combined therapy against GBM (Gong et al., 2020).

##### 2.2.1.3 Quantum dots

Quantum dots are semiconductor crystal materials ranging from two to 10 nm (Jamieson et al., 2007; Gawel et al., 2022), which can easily penetrate BBB. They have unique photophysical properties and are also excellent candidates for PTT. Quantum dots have intrinsic cytotoxicity; therefore, their administration without targeted delivery might be dangerous (Perini et al., 2020a; Gawel et al., 2022). Two types of quantum dots are widely used in GBM studies: gold quantum dots showing acceptable antitumor activity and limited cancer cells’ progression (Wahab et al., 2019) and graphene quantum dots with more biocompatibility than other quantum dots (Perini et al., 2020a). The latter proved to be an excellent carrier for chemotherapeutic agents like DOX (Perini et al., 2020a; Perini et al., 2020b). As a promising approach to drug delivery to the GBM site, cancer CM has been applied to deliver DOX-loaded graphene quantum dots (Ren et al., 2022).

#### 2.2.2 Silica-based NPs

Mesoporous silica nanoparticle (MSN) is the most popular type of silica-based NP in nano-medicine and drug delivery (Tang et al., 2012). Although MSN does not pose an intrinsic therapeutic effect, its excellent biocompatibility provides a great opportunity for drug delivery and imaging in various medical cases (Tang et al., 2012; Luo et al., 2019). Other useful properties of MSN are its favorable chemical characteristics, thermal stability, and its controlled drug delivery and release thanks to its silica component (Kazemzadeh et al., 2022). Transferrin-modified MSN has been shown to be well-performed for targeted drug delivery in GBM (Luo et al., 2019; Li et al., 2022a). Combined therapeutic approaches are much more manageable with CM coating. Li et al. designed a multifunctional drug for GBM based on MSN and erythrocyte membrane, which are further discussed (Li et al., 2022a).

As MSN is considered a nanoparticle with both silica and mesosporous components, they could simultaneously benefit from the chemical and physical stabilities of silica and useful structural cavity network of mesosporous. MSNs pose several useful characteristics making them attractive options for drug-carrying purposes in medicine. These properties include their desirable pores’ volume, their favorable loading capacities, their particles’ manageable size and shape, and their convenient way of production. Although, considerable advances have been made in developing and arrangement of MSNs for medical applications, the literature still requires more studies like qualified animal studies before MSNs can be used in the clinical setting (Kazemzadeh et al., 2022).

Besides MSN, a pure silica NP, other NPs can be mixed with silica for improved biocompatibility, stability, and sometimes a reduction in expenses (Wu et al., 2021). For example, (Wu et al.,2021) covered magnetic NPs with silica for safer thermotherapy of GBM. Moreover, gold NPs usually have a silica core (Pall et al., 2019). Moreover, considering the results of clinical studies, silica NPs might contribute to the development of future nanovaccines and theranostics. However, there are still significant barriers preventing their wide application in clinical trials. These challenges include their relatively unknown long-term adverse effects, the concerns about their safety profile when they are used chronically, the methods for reproducing them, and their useful scale-up methods (Kazemzadeh et al., 2022).

#### 2.2.3 Synthetic-polymeric NPs

The use of biocompatible and biodegradable polymers is widespread in medicine (Kapoor et al., 2015). There are several polymeric nano-carriers, from which only the most beneficial types in GBM are discussed. Polylactic-co-glycolic acid (PLGA) is an FDA-approved material for medical applications. It can carry both hydrophilic and hydrophobic drugs with prolonged secretion (Kapoor et al., 2015). PLGA is believed to be the first NP core in CM-coated NPs technology (Hu et al., 2011). It also has been applied repeatedly in GBM drug deliveries. In this regard, Ben-Akiva et al. synthesized PLGA NPs in anisotropic shapes and coated them in the Red blood cells (RBCs) membrane for more extended blood circulation (Ben-Akiva et al., 2020). In GBM, CM-coated PLGA has been loaded with DOX or docetaxel (DTX) for enhanced chemotherapy in the GBM tumor site (Chai et al., 2017; Zou et al., 2018; Liu et al., 2022b).

Besides PLGA, there are other biodegradable polymeric NPs, like polylactic acid (PLA) NP or polyethylene imine (PEI). PLA, known in medicine since 1970, has been severally applied for DOX delivery (Kapoor et al., 2015; Sun et al., 2015; Zhang et al., 2021). PEI is a widely-used nano-carrier in gene therapy, posing the ability to carry DNA. PEI-DNA nano-complex formation is based on charge interactions. For targeted delivery and to modulate this complex surface charge, CM vesicles can be helpful (Han et al., 2021).

Dendrimers are other polymeric NPs without therapeutic effects that serve as vital carriers for drugs and genes. They are known for their roles in RNA delivery. Dendrimers are considered ordered and branched scaffolds. Depending on their materials, they can have either a positive or negative charge. The positive-charged dendrimers are essential for interaction with RNA and forming complexes, making them more popular than the negative-charge ones (Stenström et al., 2018). However, dendrimers’ surfaces do not have the required qualities for drug delivery. For example, the NH_2_-terminal on their surface causes toxicity, and macrophages clear it rapidly without any specific biomarker. Polymers like PEG and bio-mimetic materials like lipids, proteins, peptides, vitamins, antibodies, or aptamers can modify dendrimers’ surfaces. The cationic dendrimers interact well with the membrane vesicle (Kong et al., 2016; Ghaffari et al., 2018). As the literature lacks studies of CM-coated dendrimers, there is a need for more research in this field.

## 3 An overview on CM-coating technology

### 3.1 CM-coating technology as an eminent NPs modifier

As discussed, advances in nanotechnology have led to much progress in medicine and pharmacology, especially in the field of oncology. Still, many people worry about overdosing on synthetic drugs. It is worth mentioning that many drawbacks hinder the utility of nano-medicine in oncology, like their significant side effects in high doses (Dehaini et al., 2016; Fang et al., 2018; Raza et al., 2021).

There are several alternatives to minimize the side effects of NPs. Many of these alternatives are based on redesigning NPs to exert less toxic effects. For instance, to diminish NPs’ interaction with cell surfaces, NPs can be designed to have negative charges, or ligands like PEG can be bound to them to inhibit their excessive protein binding (Buchman et al., 2019). Moreover, to inhibit the overproduction of ROS, adding a shell layer could be beneficial (Buchman et al., 2019). Another approach to reducing NPs toxicity is to apply a targeted delivery system. Targeted delivery can be achieved with synthetic polymers like PEG or biomimetic membrane components (Chai et al., 2017; Raza et al., 2021; Liu et al., 2022a).

Synthetic materials utilization is widely spread, and FDA approved them in 2019. However, their toxicity, low biocompatibility, and low therapeutic influences made scientists consider natural-derived agents like biomimetics for NPs surface modification (Wen et al., 2021).

The surface of biomimetic NPs is engineered to imitate the features of their biological sources. In this approach, different biomaterials can be used to cover the NPs’ surface. These biomaterials can be natural membrane protein ligands like transferrin (Ramalho et al., 2022), lipoproteins, and adhesion proteins, or synthetic biomaterials like aptamers and targeting peptides. They can also be whole membranes derived from exosomes or CMs. Different cell sources are available for this approach, and each has benefits and drawbacks that are discussed further in this article (Dehaini et al., 2016; Beh et al., 2021) Figure 2.

**FIGURE 2:**
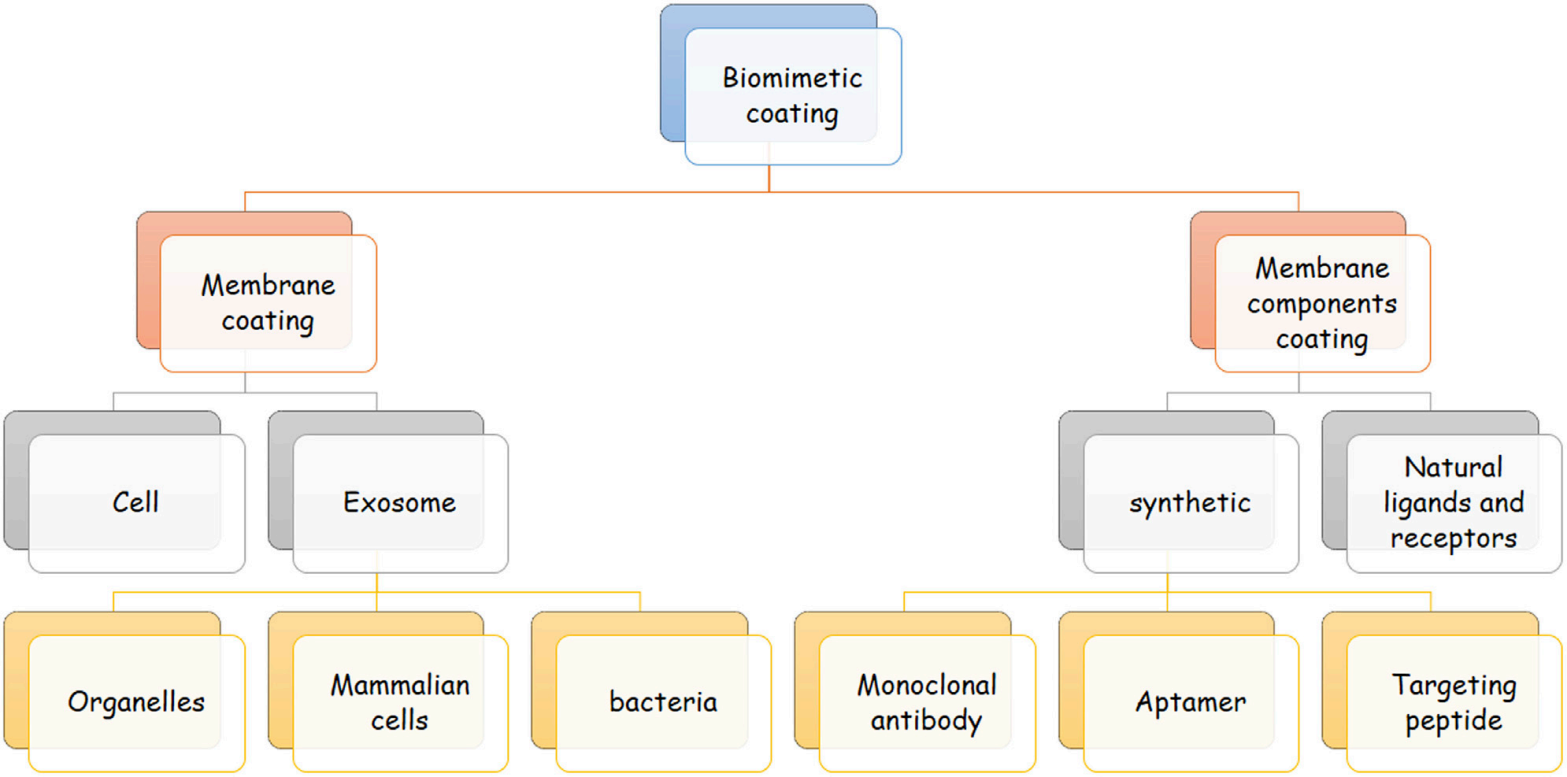
Classes of biomimetic coating. This figure demonstrates the different classes of biomimetic agents for coating nanoparticles. Biomimetic agents are either a complete membrane or separated components of a cell membrane. The membrane can be derived from exosomes and cells, and membrane components can be natural or synthetic. Exosome-derived membranes can be achieved from bacteria, mammalian cells, and different organelles. Examples of synthetic cell membranes are targeting peptides, aptamers, and monoclonal antibodies.

For various reasons, employing the whole CM for NPs coating is more worthwhile than all of the mentioned biomaterials. A whole membrane contains many structures, each having a particular responsibility. Thus, it is reasonable that NPs covered with all components necessary for cell survival circulate longer in the body than NPs coated with specific proteins and bio-structures. In other words, single CM coating is more beneficial and straightforward than multiple biomimetic membrane agent fusion (Gao and Zhang, 2015; Beh et al., 2021; Rampado et al., 2022). For example, for prolonged circulation in the body, the presence of CD47 protein on specific CMs, like erythrocyte membranes or cancer CMs prevents macrophages from attacking NP (Fam et al., 2020). On the other hand, some cell surface proteins on some cells, such as cancer cells, carry antigens and could elicit the immune response to cancer and accelerate cancer treatment (Gao and Zhang, 2015). Therefore, CM-coating technology can provide the most benefits with the least effort and costs.

### 3.2 Preparation of CM-coated NPs

**FIGURE 3:**
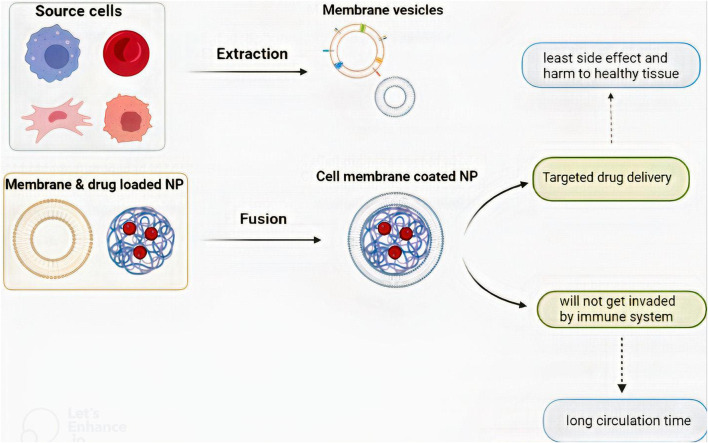
The process of CM-coated NP preparation and the benefits that CM-coated NPs provide. There are three steps to preparing a CM-coated NP: i) membrane vesicle extraction, ii) core nanoparticle construction, and iii) fusion of the membrane vesicle and core NP.

There are three steps for the preparation of CM-coated NPs: i) the extraction of the membrane vesicle, ii) core nanoparticle construction, and iii) the fusion of the membrane vesicle and core NP (Figure 3) (Zhai et al., 2017). Each of these steps varies depending on the cell source or NP type. The NP core is a nano-carrier loaded with therapeutic agents. As discussed earlier, some NPs have therapeutic effects. Also, plenty of therapeutic agents can get loaded in an NP and provide the opportunity for combined therapy which is vital in GBM treatment (Yang et al., 2020).

There are two types of extraction depending on the presence of the nucleus. After cell isolation and purification, nucleus-free cells like erythrocytes must be lysed so the cell contents exit. Cell lysate can be chemical with hypotonic features (Mendes et al., 2020) or mechanical with the freeze-thaw process (Zhai et al., 2017; Zou et al., 2018). Membrane vesicle extraction from nucleated cells requires a more complicated process. An extra discontinued sucrose gradient centrifugation must be done after cell lysate to remove organelles and nuclei in these cells. They also must be washed with buffers like phosphate-buffered saline (Zhai et al., 2017; Fan et al., 2021a). Consequently, membrane extraction is a combination of lysis and extraction. The process must be done at 4°C with protease inhibitors (Zou et al., 2020).

There are several ways to fuse membrane vesicles and NPs (Zou et al., 2020). They can be either extruded with the help of plenty of nanoscale sporous membranes (Gao et al., 2016a) or sonicated with a sonicator (Hu et al., 2015). Microfluid chips can also be used for the electroporation technique (Rao et al., 2017). Before fusion, the amount of membrane and NPs needed should be precisely calculated so the material waste becomes minimum (Zou et al., 2020). After preparation, various tests could be conducted to evaluate if the CM-coated NPs were correctly engineered. Transmission electron microscopy illustrates the core and coating. Moreover, *via* polyacrylamide gel electrophoresis protein separation, researchers can evaluate if membrane proteins are unharmed (Han et al., 2019; Johnson et al., 2022).

Moreover, it has been shown that averagely only 0.7% of injected NPs could reach the tumor site (Wilhelm et al., 2016). On the other hand, studies have shown that CM-coating technology leads mostly to the formation of partially-coated NPs. In other words, more than 90% of NPs were shown to be only partially coated with CM after they were affected by mechanical forces resulting from ultrasonication. These partially-coated NPs have some significant drawbacks compared to completely-coated ones. Though partially-coated NPs demonstrated some efficacies in internalization in targeted cells *in vitro,* it has been shown that only 40% of them can enter the targeted cells to initiate their effects *in vivo* (Liu et al., 2021a). These disadvantages make scientists consider various methods for converting NPs partially-coated to completely-coated ones. Although the precise underlying mechanism for producing partially-coated NPs is not elucidated, a well-conducted research by Liu et al., demonstrated that limited CM fluidity is the major factor responsible for the failure of NP fusion with surrounded CM resulting in partially-coated NPs. Based on this concept, they introduce a novel method for full coating of NPs. This method includes introducing external phospholipids to enhance the CM fluidity, facilitating the ultimate fusion of lipid patches to NPs. With the mentioned approach, the rate of NPs with full coating reaches 23% which is significantly higher than previously-designed full coating approaches (6%). Moreover, full coated-NPs achieved by this technique showed a significant better tumor targeting and accumulation properties (Liu et al., 2022c).

### 3.3 Introduction to membrane sources and their characteristics

Despite all the efforts that scientists have made to imitate the structure of CMs, nothing has been able to replace natural membranes completely. Therefore, the best structure for coating NPs is using natural cells. Natural cells derived from the human body are less likely to activate immune responses (Ben-Akiva et al., 2020). The following section reviews candidate cell types for CM-coating technology in cancer treatment and GBM therapy. An overall preview of these cells’ characteristics is listed in Table 2.

**TABLE 2:** An overview of different cell membrane characteristics.

Cell membrane type	Inherent targeted delivery	Penetration through BBB	Immune response activation	CD47 marker
Erythrocyte	×	✓	×	✓
Leukocyte	✓	✓	✓(mostly)	✓
Cancer cell	✓	✓	✓	✓
Mesenchymal stem cell	×	✓	×	✓

#### 3.3.1 Erythrocytes

RBCs have excellent qualities for camouflaging NPs and their delivery inside the body. These properties include appropriate size, shape, essential membrane proteins, immune escape ability, and relatively long circulating time (Tzeng et al., 2019; Raza et al., 2021). The idea of using RBCs’ membrane as a delivery vesicle was first introduced in 1994. Also, the first study on CM-coating technology was done with RBCs as the source cell (Chugh et al., 2021). The RBC membrane-coating technology has been applied to cancers like pancreatic cancer, breast cancer, colon cancer, lymphoma, leukemia, and GBM (Yang et al., 2019; Chugh et al., 2021).

In GBM, RBC is a popular choice for therapeutic agent encapsulation. For instance, RBC membrane-coated NPs have been designed for the delivery of therapeutic agents, including famous DOX, PLGA (Chai et al., 2017; Ben-Akiva et al., 2020; Liu et al., 2022b), gene therapy agents like DNA and RNA (Liu et al., 2020a; Han et al., 2021; Li et al., 2022a), and agents like indocyanine green (ICG) for synergic GBM therapy (Wang et al., 2020; Li et al., 2022a). RBC membrane-coated NPs could also be used for toxin removal alongside cancer therapy. RBC vesicles can absorb toxins. With the core of some unique NPs, like PLGA, they can absorb bacterial toxins and prevent hemolysis (Ben-Akiva et al., 2020; Liu et al., 2020b).

No studies have been conducted to accurately measure and compare RBC with other source cells in CM-coating drug delivery. Still, several studies indicated that RBC membrane could decrease side effects and toxicity in high doses as it increases drug uptake in the targeted tissue and antitumor activity (Yang et al., 2019). Also, the flexibility of the RBC membrane allows for the transport of anisotropic NPs delivery (Ben-Akiva et al., 2020).

The erythrocyte membrane does not have any direct interaction with GBM cells. Hence, they naturally cannot target tumor cells. For a targeted drug delivery system, the RBC membrane must get modified with another biomimicking agent, like targeting peptides (Li et al., 2022a) or aptamers (Liu et al., 2022b).

#### 3.3.2 Leukocytes

For various reasons, white blood cells (WBCs) can be a proper candidate for the delivery and protection of therapeutic NPs. First, they can provide targeted drug delivery. Every tumor, including GBM, can induce hypoxia and inflammation in their environments. These conditions chemotactically attract WBCs like a magnet. Hence, WBC membrane-coated NPs can reach tumors rapidly. WBCs also show their property in long-circulating time (Lewis and Pollard, 2006; Choi et al., 2007; Gao et al., 2016b; Oroojalian et al., 2021).

**FIGURE 4:**
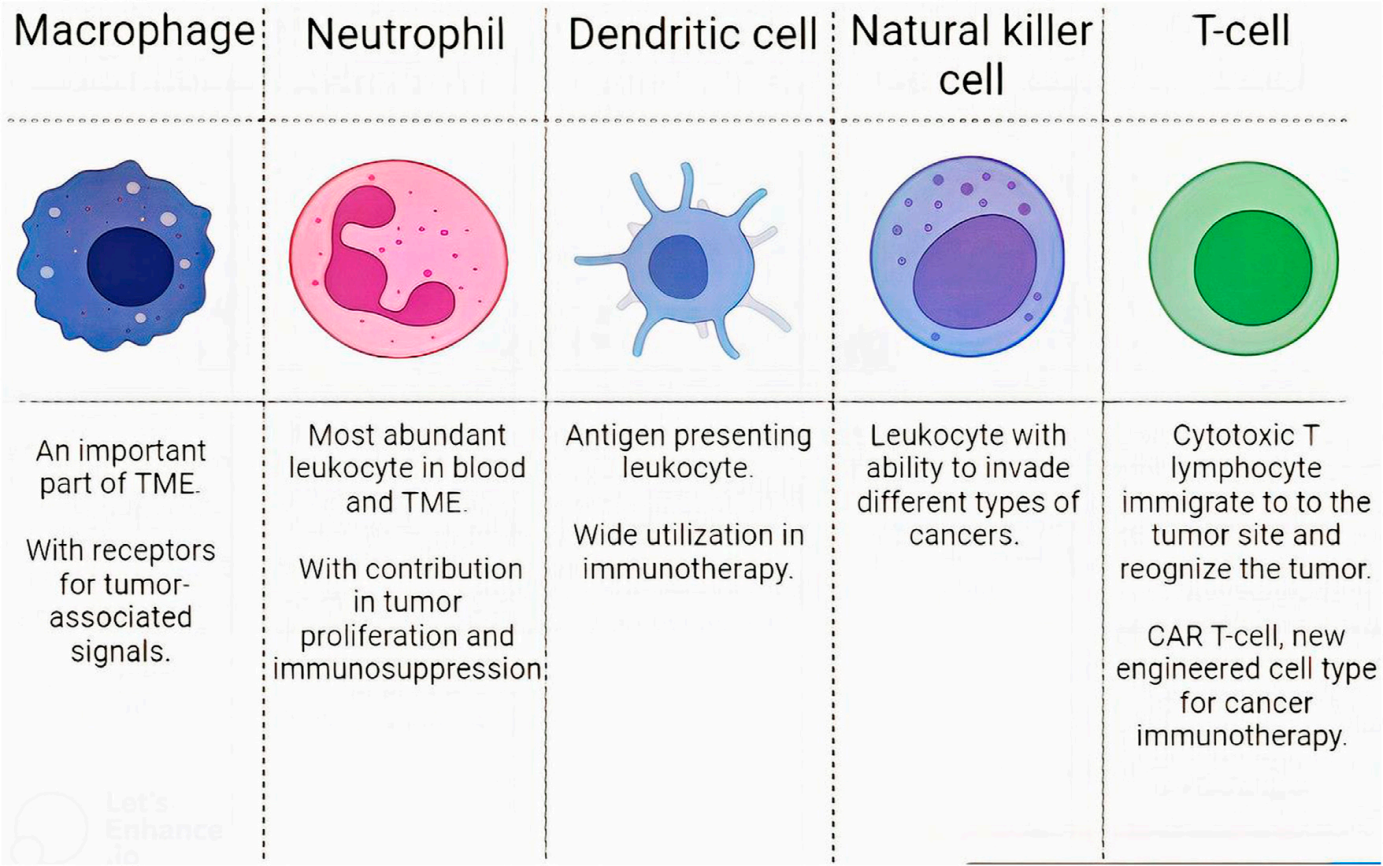
Leukocytes characteristics. The characteristics of different WBC subsets in designing CM-coated NPs.

Although some barriers exist to this targeted drug delivery, such as GBM immunosuppressive microenvironment, many studies have successfully tested WBC membrane-coated NPs in pre-clinical stages (Antunes et al., 2020; Wu et al., 2021). In this regard, WBCs like T-lymphocytes, dendritic cells (DCs), macrophages, natural killer cells (NKs), and neutrophils have been evaluated. We explain them and compare their capabilities (Figure 4).

##### 3.3.2.1 Macrophages

Macrophages are one of the most important monocyte-derived immune cells, and Microglia are specialized macrophages that reside in the CNS (Heidari et al., 2021a). These cells constitute a critical part of the tumor microenvironment (TME). In this regard, macrophages residing in the tumor microenvironment are termed tumor-associated macrophages (TAMs). TAMs in GBM are either microglia or bone marrow-derived macrophages (Ochyl and Moon, 2019; Heidari et al., 2021b; Buonfiglioli and Hambardzumyan, 2021). Monocytes, bone-marrow-derived macrophages, and microglia can respond to tumor-associated signals by specific receptors on their surfaces and thereby can be recruited to the TME (Fan and Jiang, 2020). Some examples of the specific receptors absorbing macrophages to the tumor site are C-C chemokine receptor 2, vascular cell adhesion molecule 1, and intercellular adhesion molecule 1 (Gong et al., 2020). Hence macrophages’ membrane may act as an excellent drug bearer to the intractable GBM.

Macrophage-associated drug delivery to GBM is not only theoretical. In one study, zoledronate (ZOL) NPs wrapped in microglia membrane performed better than bare ones in GBM orthotopic mice model (Liu et al., 2020a). In another study, a dual-membrane based on macrophages and neutrophil membranes was developed for targeted delivery of NPs against glioma with promising results (Yin et al., 2022).

##### 3.3.2.2 Neutrophils

Neutrophils are the most abundant leukocyte in the peripheral blood and constitute a significant part of TME (De Leo et al., 2020). Their accessibility to TME give them the potential to be used as a cloak for therapeutic NPs. Results of several studies indicate that neutrophil membrane-coated NPs can successfully pass through BBB and deliver their contents to the targeted cell. Up to now, mesoporous Prussian blue enzyme and paclitaxel have been successfully delivered to the mice’s brains (Xue et al., 2017; Feng et al., 2021). Also, a dual neutrophil macrophage membrane has been designed, as discussed in the previous part (Yin et al., 2022).

Though neutrophil membrane-coated NPs can reach the tumor site successfully, it must be considered that as GBM progresses, the infiltration of neutrophils in its TME is upregulated. Meanwhile, neutrophils promote tumor proliferation and immune suppression. In this regard, patients receive medicines to prevent the migration of neutrophils into the TME. These medicines can also affect CM-coated NPs (Jin et al., 2019). Consequently, neutrophil-coated NPs might show promising results in controlled pre-clinical stages, but they might not be the best choices in the clinic.

##### 3.3.2.3 DCs

DCs are the most critical antigen-presenting leukocytes that activate T-lymphocytes using their peptide-bound major histocompatibility complex (MHC) on their surfaces (Fucikova et al., 2019; Gong et al., 2020). The use of DCs in cancer is mainly restricted to the delivery of therapeutic agents and immune therapy (Tapeinos et al., 2019a; Lee et al., 2022). They lack receptor that targets cancer cells, so they can act better in immunotherapy than in drug delivery. In one study, they were used for T-cell activation (Ochyl and Moon, 2019). However, there are currently no feasibility studies on DC or NK membrane-coated NPs in the GBM model.

##### 3.3.2.4 Natural killer cells

NKs are other immune cells that can invade various cancer cells (Chiossone et al., 2018). Their membrane has been used for targeted drug delivery to breast cancer (Pitchaimani et al., 2018). They were also designed for biomimetic nanorobots delivery to the brain regions for GBM treatment (Deng et al., 2020). NKs do not have specific antigen receptors for GBM, but they have receptors that identify tumor cells like NKG2D, NKp44, NKp46, NKp30, and DNAM (Gong et al., 2020). Hence, their membrane is reported to have the ability for targeted drug delivery through BBB. Hence, they may be used in future studies in GBM treatment and in nano-drug delivery (Gong et al., 2020).

##### 3.3.2.5 T-cells

T-lymphocytes play a vital role in tumor recognition and destruction (Boon and van der Bruggen, 1996). Cytotoxic T lymphocytes (CTLs) can migrate to the tumor site and recognize the tumor with the T-cell receptors (TCRs) (Kang et al., 2020). With their help, The importance of T-cells in cancer therapy has brought many innovative ideas for faster and more robust responses (Themeli et al., 2013). These innovations appeared in the field of CM-coated NPs. TCR can recognize and bind to special tumor cells. This T-cell ability can provide a tremendous targeted delivery as it was used for targeted PLGA delivery to melanoma cells and melanoma immunotherapy (Yaman et al., 2020b; Kang et al., 2020).

A novel therapeutic approach for cancer immunotherapy based on TCR is chimeric antigen receptor (CAR) T-cells. CAR T-cells have been engineered to recognize and attack tumor cells specifically. A combination of CAR T-CMs for specific targeting and nano-based drugs for a better impression can be influential. This approach has been applied to animal models in carcinoma and myeloma but has never been tested for GBM (Ma et al., 2020; Van der Schans et al., 2020).

#### 3.3.3 Cancer cells

Many studies have used cancer CM-coated NPs for their numerous advantages. First, cancer cells have homotypic targeting, so cancer membrane-coated NPs can easily find and bind to similar cells. The most critical biomarkers on GBM cells for homotypic recognition are cadherin-2, stromal cell-derived receptor 1, and β-catenin (De Pasquale et al., 2020). Second, they can circulate quickly through the body and present tumor antigens to enhance immune system responses. Third, cancer NPs coated with CM can evade macrophages and benefit from a longer circulation time due to their immune evasion property. Moreover, the short cell cycle of cancer cells makes the cultivating and membrane extraction process much faster and easier (Liu et al., 2020b; Zou et al., 2020; Chugh et al., 2021; Guo et al., 2022).

Numerous studies have designed GBM CM–coated NPs and have evaluated them in mice models or *in vitro*. The GBM membrane has been designed to deliver chemotherapeutic drugs (Tapeinos et al., 2019a; De Pasquale et al., 2020; Fan et al., 2021a; Fan et al., 2021b; Ren et al., 2022; Zou et al., 2022), gene therapy agents (Han et al., 2021), and photosensitizers for photo-based synergic therapies (Wang et al., 2020; Lu et al., 2022; Ren et al., 2022). They are also considered efficient antigen-presenting cells and could increase T-cells, DCs, and inflammatory factors (Zou et al., 2018; Jin et al., 2019; Wu et al., 2021; Wang et al., 2022). The details of each of these studies are further elucidated in this review.

#### 3.3.4 Mesenchymal stem cells

Mesenchymal stem cells (MSCs) are another cell group that can immigrate to tumor sites. This ability has brought much attention to MSCs in cancer therapy. To improve the targeting potential of mesenchymal stem cells, they can also get modified with antibodies, targeting peptides, and special receptors. Some receptors like chemokine (C-X-C motif) receptor 4 (CXCR4) and C-C chemokine receptor type-1 (CCR-1) have successfully been modified on the MSC surface for better inflammation and tumor site tracking (Park et al., 2015; Zou et al., 2020). Drug delivery is one of the most prominent applications of MSCs in cancer therapy. A study demonstrated that MSC nano-drug carriers perform better than Individual MSCs, which is routine. In the same study, MSC could easily penetrate mice BBB (Suryaprakash et al., 2019). Although more pre-clinical studies are necessary for this area, MSC membrane-coated NPs may be an ideal candidate for targeted NP delivery.

#### 3.3.5 Other sources

In addition to the mentioned cells, other sources like platelets or bacteria have been applied for CM-coated NP drugs. However, it is unclear whether these sources can be applied successfully for GBM treatment considering some features, like the ability to penetrate through BBB (Buonfiglioli and Hambardzumyan, 2021; Sun et al., 2022). It is also worth mentioning that besides the plasma membrane, the organelle’s membrane, like the nucleus or mitochondria membrane, can be efficiently applied for NP-targeted gene and drug delivery (Nurunnabi et al., 2019). Also, new studies are considering combined membranes as a new and promoted generation of CM vesicles with higher efficacy and lesser drawbacks (Guo et al., 2022).

Finally, besides membranes derived from complete cells, exosomes could also help coat NPs. Exosomes have been successfully applied for cancer treatment and drug delivery to the brain (Samanta et al., 2018). For instance, (Zhang et al.,2021) successfully utilized exosomes from endothelial cells to deliver DOX for GBM treatment. The significant difference between the exosome membrane and plasma CM is the presence of the CD47 marker on most of the types of plasma membranes, which makes its blood circulation longer. Besides, various markers on the plasma membrane that are not available on exosomes make the cellular uptake of CM-coated NPs easier. Meanwhile, we should not ignore the broad utilization of exosomes when it comes to brain disorders and passing through BBB (Lins et al., 2022; Salarpour et al., 2022). However, exosome coating is newer than CM-coating technology and requires more studies and investigations.

Exosomes are classified as a group of extracellular vesicles ranging from 30–150 nm containing different materials including protein, lipid, and nucleic acid, depending on their origin cells and having significant roles in intercellular communication and also treating a wide range of diseases (Broekman et al., 2018; Pegtel and Gould, 2019; Zhai et al., 2020). Moreover, they pose the advantage of crossing BBB, making them good choices for treating brain diseases (Li et al., 2022b). Because of their BBB penetration features, they could help design novel therapeutic approaches targeting GBM. However, despite the advantage of crossing BBB, only a tiny proportion of injected exosomes could reach the brain and tumor sites because of their primary distribution in the spleen and liver. Therefore, NPs could serve as a valuable means for adequately enriching exosomes to the GBM cells. In this regard, combining the desired drug delivery and tumor-targeting properties of magnetic NPs (Fe_3_O_4_ NPs) with BBB penetration and Small interfering RNA (siRNA)-carrying properties of engineered exosomes led to significant treatment efficacy in the mice model of GBM.

On the other hand, brain-derived exosomes (BDEs) can be released by all CNS cells and have roles in establishing communication between neurons, glial cells, and their surrounding tissues. These brain-derived exosomes could play roles as diagnostic biomarkers giving clues on different brain diseases like GBM. Moreover, because of the BBB penetrating features of BDEs, they could be useful for designing novel therapeutic approaches targeting GBM (Salarpour et al., 2022).

## 4 Therapeutic approaches for GBM treatment *via* CM-coated NPs

NPs loaded with other therapeutic agents can enhance the efficacy of cancer therapy (Tapeinos et al., 2019a). Various treatments are available for GBM, including chemotherapy, radiotherapy, immunotherapy, and gene therapy (Janjua et al., 2021). Individual treatments may not be sufficiently practical due to their short response duration and lack of targeted delivery. Moreover, the natural impermeability of BBB and tumor recurrence and resistance to drugs induced by GSCs question the efficacy of single agents for combating brain cancers. The solution is to use combined therapies in GBM, which provide opportunities to overcome the resistance and recurrence caused by GSCs, as is further discussed in this section. The combination of NPs and drugs and coating them with CM for facilitating their pathway through BBB and targeted drug delivery has recently attracted much attention to overcome barriers in GBM treatment. Accordingly, several studies have evaluated the efficiency of this novel method. The available applications of CM-coated NPs for GBM treatment are listed in Table 3.

**TABLE 3:** A comparison among therapeutic approaches for GBM treatment.

Method	Therapeutic agents	Method path	CM-coated NP application
Chemotherapy	Chemotherapeutic drugs, e.g. TMZ-DOX-ZOL Zou et al. (2022)	Eliminating the tumor progression by causing damage in GSCs or inhibit the proliferation and metastasis of GSCs Minniti et al. (2009); Trejo-Solís et al. (2018).	• Co-delivery of TMZ with other chemotherapeutics for TMZ-resistance cancer Zou et al. (2022)
			• Targeted delivery for fewer side effects of chemotherapeutics.
			• Synergic chemotherapies Ren et al. (2022)
Immunotherapy	Immunotherapies drugs, e.g. IDO, JQ1 Liu et al. (2021b); Wang et al. (2022) ZOL Qiao et al. (2021), DTX and antigen Jin et al. (2019)	Remodeling TIME Ma et al. (2018), changing TAM populations Liu et al. (2021b); Wang et al. (2022), Enhancing immune responses against tumor Sanatkar et al. (2022)	• Co-delivery of immunotherapeutic agents with other therapeutics Qiao et al. (2021).
			• Antigen presenter Jin et al. (2019)
Gene therapy	DNA-RNA Cross and Burmester (2006); Kwiatkowska et al. (2013)	Killing cancer cells by activating suicidal genes, moderating overexpression of unregulated genes Cross and Burmester (2006); Kwiatkowska et al. (2013)	• Delivery of NAs to the tumor site Sung and Kim (2019); Han et al. (2021); Scully et al. (2022)
Synergic phototherapy	Photosensitizers like ICG or chlorine-6 Fong et al. (2018); Hak et al. (2021)	Creating ROS or extra heat to kill cancer cells Fong et al. (2018); Shi and Sadler (2020); Hak et al. (2021)	• Delivery of phototherapeutic with other agents for synergic therapy Lu et al. (2022)
			• NPs like magnetic NPs are photosensitizer Yang et al. (2012)

TMZ, Temozolomide; DOX, doxorubicin; ZOL, Zoledronate; GSC, GBM stem cell; IDO, indoximod; DTX, docetaxel; TIME, tumor immunosuppressive microenvironment; TAM, tumor-associated macrophages; ICG, Indocyanine green; ROS, reactive oxygen species; NAs, Nucleic acids; NPs, Nanoparticles; DNA, Deoxyribonucleic acid; RNA, Ribonucleic acid.

### 4.1 Chemotherapy

Chemotherapy is one of the most common treatment approaches for various cancers. In 1946 for the first time, chemotherapy was used for lymphoma treatment (Joensuu, 2008). The conventional treatment of GBM includes chemotherapy with surgery and radiotherapy (Room, 2016). Chemotherapeutic agents for GBM treatment eliminate the tumor progression with two mechanisms; they either damage GSCs and trigger apoptotic pathways or prevent the proliferation and metastasis of GSCs by inhibiting growth factors (Minniti et al., 2009; Trejo-Solís et al., 2018).

#### 4.1.1 Temozolomide-resistance chemotherapy

TMZ is an FDA-approved drug for GBM chemotherapy. TMZ causes DNA damage by methylation of the O6 position of guanine bases (Room, 2016; Rajaratnam et al., 2020). Chemotherapy with TMZ has been effective in increasing the survival rate of GBM patients but could not cure the disease entirely due to the delivery and targeting challenges induced by the tumor (Trejo-Solís et al., 2018). In addition, approximately half of GBM patients suffer from TMZ-resistant tumors, which may be innate or acquired. Some abnormalities in the genetic profile of GBM patients could lead to innate TMZ resistance. On the other hand, the natural characteristics of GSCs are responsible for the acquired form of this resistance (Lee, 2016; Chien et al., 2021).

Cisplatin (CDDP) is another antitumor drug administered in GBM. This drug could inhibit TMZ resistance of the tumor; therefore co-administration of cisplatin with TMZ might be beneficial (Mishima et al., 2000; Silvani et al., 2004). In this regard, Zou et al. applied GBM-CM and PH-sensitive magnetic NPs for targeted delivery of TMZ and CDDP. This new combined strategy in a mice model with an orthotopic GBM tumor exerted more promising effects than simple-agent chemotherapy. Moreover, it did not show any side effects (Zou et al., 2022). In this regard, various properties of GBM-CM overcome the challenges previously hindering the sufficient efficacy of this combined therapy. These challenges included limited BBB penetration, weak targeting of GBM cells, and systemic adverse effects. In this context, as GBM-CM mimics the features of GBM cells, it can easily penetrate BBB. Considering the concept of homotypic membrane-membrane recognition, GBM-CM can efficiently recognize and target GBM cells without exerting notable side effects on surrounding tissues (Zou et al., 2022).

ZOL is another chemotherapeutic drug for TMZ-resistant GBM. ZOL inhibits farnesyl pyrophosphate synthetase regulation and activates the caspase-dependent apoptotic pathway. However, it cannot be used for brain tumors without a supportive agent facilitating its pathway through BBB. In this regard, Qiao et al. designed a biomimetic drug delivery system based on a microglia membrane to deliver ZOL-loaded polypropylene glycol dithiol propionate NPs to an orthotopic TMZ-resistant GBM mice model. The results indicated that NPs encapsulated in CM had more cellular uptake than bare NPs. The targeted delivery was successful. Hematoxylin and eosin (H&E) staining did not indicate any damage to other mice’s organs; on the other hand, the H&E staining of intact brain tissues in the orthotopic TMZ-resistant GBM mice model demonstrates a significant reduction of tumor size. Hence, the effects of CM-coated ZOL NPs in the tumor size reduction were more significant than free ZOL or ZOL-NPs (Qiao et al., 2021). These results indicate the beneficiary roles of coating drug-carrier NPs with microglia CM, which has enhanced biocompatibility and reduced toxic effects on other organs. In this study, the mentioned microglia CM-coated nanoparticle was efficiently recruited to the GBM tumor sites using the signals between GBM and microglia CM, including C-X3-C motif (CX3C) ligand 1 (CCL1) and CX3C receptor one or colony-stimulating factor 1 (CSF-1)/CSF-1 receptor (Qiao et al., 2021).

On the other hand, the result of a study that used MSCs as the source of CM for CM-coated NPs in both orthotopic rats and *in vitro* demonstrated that, like GBM CM, MSCs could effectively cross the BBB and migrate to the tumor area by the interaction of their receptors, namely CCR2 and CXCR4 with GBM receptors and also through the secretion of chemoattracting factors like monocyte chemoattractant protein-1/CCL2 by GBM cells. However, the histological evaluation of GBM tumors treated with MSCs in this study did not indicate effective results as the group treated with MSCs showed tumor development and enhanced invasiveness, which might be due to the secretion of exosomes by MSCs, limiting their wide application in CM-NP technology for carrying chemotherapeutic drugs like TMZ to the tumor (Pavon et al., 2018).

#### 4.1.2 DOX chemotherapy

DOX is a well-known chemotherapeutic drug posing tremendous anticancer effects. It restricts cell division by disrupting topoisomerase II activity. Like many other agents, DOX should not be used alone in GBM treatment due to its poor ability to penetrate the BBB. However, direct intra-tumor injection of DOX could efficiently improve its access to the tumor site. Several studies suggested loading DOX in NPs, especially PLGA NPs, to facilitate its pathway through BBB (Steiniger et al., 2004; Malinovskaya et al., 2017; Maksimenko et al., 2019).

DOX also demonstrated excellent efficacy when loaded in boron nitride nanotubes (BNNTs). In this regard, De Pasquale et al. showed in an *in vitro* study that combined DOX, BNNTs, and GBM CMs developed effective nanoplatforms for GBM therapy (De Pasquale et al., 2020). Homotypic membrane-membrane recognition, the biocompatibility of BNNTs, and the anticancer effects of DOX could lead to effective BBB penetration, cell targeting, and antitumor activity. The BBB-crossing potential of CM-coated BNNTs was more prominent than PEG-coated BNNTs *in vitro* models. In this regard, in a dynamic model of BBB, CM-coated BNNTs showed 15 times better crossing tendency than PEG-coated BNNTs. The excellent efficiency of this nanoplatform can be attributed to the coated CM proteins facilitating homotypic recognition and specific targeting of GBM cells. Hence, the cell-membrane camouflaging property of CM-NPs could play a significant role in overcoming the usual biological barriers existing in the way of NPs’ application in GBM patients, including intratumoral heterogeneity, limited BBB penetrance, and therapy resistance resulting from GSCs features. Accordingly in this study, optical emission spectroscopy proved that the approach was successful in the homologous targeting of GBM cells. Likewise, no significant side effects were reported in healthy cells (De Pasquale et al., 2020).

DOX with PLGA NPs were also loaded in an engineered RBC membrane. For better brain targeting delivery, (Chai et al.,2017) developed a new idea in CM-coating technology. They modified the RBC membrane by adding a neurotoxin-derived peptide, CDX, to its outer surface. CDX is a candoxin-derived peptide, binding with high affinity to nicotinic acetylcholine receptors in the brain’s endothelial CMs. The fluorescent imaging of both primary brain endothelial cells *in vitro* and orthotopic GBM mice model indicated a more efficient cellular uptake in CDX-RBC membrane NP than normal RBM membrane NP. Furthermore, the transcytosis of CDX-modified NPs across BBB was shown to be enhanced. This innovation increased cellular uptake and made the drug delivery system for DOX more efficient. The survival curve of four groups of orthotopic GBM mice injected with saline, free DOX, DOX-loaded RBC NPs, and DOX-loaded CDX-RBC NPs, proved a better antitumor efficacy of CDX-RBC NPs than others. Furthermore, the mice treated with CDX-RBC and RBC NPs did not indicate any cardiac malfunction or weight loss as side effects, while free DOX-treated mice showed systolic dysfunction and significant weight loss (Chai et al., 2017). In this context, RBC CM imitated the natural CM functions facilitating its pathway through BBB, and when incorporated with CDX, the complex could achieve high targeting ability (Chai et al., 2017).

Aptamers are single-strand nucleic acids that can bind to their supplementary three-dimensional structures. Aptamers can target proteins, cells, viruses, peptides, polysaccharides, nucleic acids, and other organic and inorganic molecules (Cesarini et al., 2020; Fu and Xiang, 2020). In this regard, aptamers could be perfect agents for cancer-targeted drug delivery. Another DOX study used a modified RBC for GBM treatment in an orthotopic GBM mice model. Based on the fluorescent microscope and flow cytometry results, the aptamer-modified RBC membrane caused more cellular uptake. Moreover, the aptamer modification increased the survival rate of the intracranial GBM-bearing mice model from 15.5–23 days. Also, the fluorescence imaging results demonstrated the aptamer-modified RBC-membrane coated NPs deep into GBM tissue that successfully accumulated in heterogeneous tumor cells (Liu et al., 2022b). This result indicates that CM-NPs could effectively conquer the intratumoral heterogeneity of GBM, which challenged previous treatment efforts.

#### 4.1.3 Synergic chemotherapies

The combination of drugs and NPs in the CM-coating strategy can bring an excellent opportunity for synergistic therapies. For example, (Ren et al.,2022) coated DOX with graphene quantum dots (GQDs) in the GBM membrane. GQDs can cause a photothermal effect. In NPs-assisted PTT, laser radiation can cause hyperthermia and destroy cancer cells. In this study, the co-encapsulation of NPs for PTT and chemotherapeutic drug (DOX) with the help of a homotypic cancer CM was successful. Laser stimuli, which are necessary for PTT, destroyed the membrane in the tumor site and caused a rapid release of Dox. Hence, this study introduced a new generation of drugs in GBM (Liu et al., 2020c; Ren et al., 2022).

The synergic effect of chemotherapeutic agents and hyperthermia is termed termochemotherapy (Rani et al., 2020). Tapeinos et al. synthesized nanocubes composed of magnetite (Fe_3_O_4_) and manganese dioxide (MnO_2_). MnO_2_ releases heat and increases the intracellular temperature in a reaction with H_2_O_2_. For the first time in literature, ROS contributed to increasing intracellular heat. The drug-loaded nanocubes also enhance cancer cell destruction. Under AMF, the amount of apoptotic and necrotic cells increased. The nanocubes with a chemotherapeutic drug tested in phases I and II of clinical trials were coated with GBM-membrane. The GBM membrane encapsulation brought up to the 75% NPs passage through the monolayer epithelial cell BBB model *in vitro*. In addition, the CM-coated magnetic NPs could cross dynamic BBB and accumulate under the magnetic field. The result of the study was promising *in vitro*. However, more *in vivo* experiments are necessary (Tapeinos et al., 2019a).

Yanjie Liu et al. encapsulated TMZ with OTX015 (OTX) for combined chemotherapy and immunotherapy. OTX can inhibit the programmed cell death ligand, an overexpressed gene in cancers that disrupts T-cells functions. The process can reduce the GBM immunosuppressive microenvironment. OTX is a helper for TMZ and increases cell sensitivity to TMZ by disrupting the DNA damage repair response. Hence, the co-encapsulation of OTX and TMZ not only enhances treatment by the synergic effect of immunotherapy and chemotherapy but enhances the therapeutic effect of TMZ and reduces TMZ resistance. With the help of the RBC membrane modified with apolipoprotein E peptide, OTX and TMZ NPs penetrate through BBB and targeted GBM cells in an orthotopic GBM-bearing mice brain (Liu et al., 2022d).

CM-coated NPs used as chemotherapeutic agents are diverse and listed in Table 4.

**TABLE 4:** A summary of CM-coated NP studies in chemotherapy of GBM.

Author/reference	Cell membrane source	Drug contents	Nanoparticle	Experimental model	Achievements
Zou et al. (2022)	GBM cell	CDDP and TMZ	PH-sensitive magnetic NP	*In vitro* and orthotopic TMZ-resistant GBM mice model	-Decrease in side effects
					-Indicates better results than single therapy
Qiao et al. (2021)	Microglia cell	ZOL	PDP NP	*In vitro* and orthotopic TMZ-resistant GBM mice model	-Successful targeted delivery
					-Improvement in hypoxia and suppressive microenvironment improvement.
					- Reduction in GBM proliferation
De Pasquale et al. (2020)	GBM cell	Dox	BNNT	*In vitro* model	-Easy penetration through *in vitro* BBB model
					-Homologous targeting
					-Antitumor activity against GBM cells with no significant harm to healthy cell
Chai et al. (2017)	Modified RBC cell	Dox	PLGA	*In vitro* and orthotopic GBM mice model	-Easy penetration through BBB both in mice and *in vitro*
					-More advanced targeted delivery with CDX peptide
					-Significant antitumor and anti-proliferation effect.
Liu et al. (2022b)	Modified RBC cell	Dox	PLGA	*In vitro* and intracranial GBM-bearing mice model	- More advanced targeted delivery with aptamer
					-Significant antitumor and anti-proliferation effect and increase in survival rate.
Carvalho et al. (2019)	Glioma Cell	Dox	GQDs	*In vitro* model	-Homologous targeting
					-Significant increase in cellular uptake
					-Fast release of DOX
					-Increase in cell apoptosis
Tapeinos et al. (2019a)	GBM cell	sorafenib	Fe_3_O_4_ and MnO_2_ nanocubes	*In vitro* model	-Homologous targeting
					-Increase in cell apoptosis and necrosis
					-The first literature review with an increase in intracellular temperature
Liu et al. (2022d)	Modified RBC	TMZ and OTX	Acetal-dextran (pH-sensitive NP)	*In vitro* model and orthotopic mice model	-Synergic chemotherapy and immunotherapy
					-reduction in tumor resistance to TMZ

NP, Nanoparticle; GBM, Glioblastoma multiform; PH, potential of hydrogen; CDDP, Cisplatin; ZOL, Zoledronate; BBB, Blood-brain barrier; PDP, Polypropylene glycol dithiol propionate; DOX, Doxorubicin; BNNT, Boron nitride nanotubes; CDX, Candoxin-derived peptide; PLGA, Polylactic-co-glycolic acid; GQD, Quantum dot; Fe_3_O_4_, Iron (II, III) oxide; MnO_2_, Manganese (IV) oxide.

### 4.2 Immunotherapy

Immunotherapy in oncology refers to actions that strengthen the body’s immune system responses to cancer cells (Sanatkar et al., 2022). Immunotherapy is more effective and less toxic than chemotherapy for cancer treatment (Tan et al., 2020b; Gong et al., 2020). However, despite several advancements in immunotherapy, it has not led to a sufficient level for treating GBM patients (Abadi et al., 2021). The tumor immunosuppressive microenvironment (TIME) has a vital role in cancer immunotherapy. Some malignancies, like brain tumors, have a tumor TIME, which exerts many challenges in brain tumors, especially in treating GBM (Ma et al., 2018).

Wang et al., (2022) developed a novel approach to remodel TIME in GBM patients using CM-coated NPs technology. They combined Cu2-xSe NP with indoximod (IDO) and JQ1 and then coated them with a tumor CM. This compound affects TIME in several ways. Each of the materials used in the drug has a significant role. Cu2-xSe NPs can reduce microenvironment hypoxia, increase M1-phenotype macrophages, and reduce M2-phenotype macrophages in the tumor environment. As M1-phenotype has antitumor properties, and the M2-phenotype is a tumor promoter; this conversion is crucial in the TIME remodeling. IDO and JQ1 are immunotherapeutic drugs that have been studied in clinical trials. The targeted delivery of these drugs in lesser doses reduced their toxicity and increased their impacts. The new compound was tested *in vitro* and in an orthotopic mice model. The effect of immunotherapy was evaluated with immunofluorescence imaging and flow cytometry analysis. They indicated more CD8^+^ T-cells in the tumor site and the spleen of the orthotopic GBM-bearing mice model than in the control group. Tumor necrosis factor-alpha was also increased in the tumor site. The concentration of DCs in the lymph nodes also increased. As expected, M1-macrophages increased, and M2-macrophages decreased (Liu et al., 2021b; Wang et al., 2022). ZOL, a chemotherapeutic drug for TMZ-resistance GBM, can have a similar effect on TAMs. In this regard, Qia et al. designed CM-coated ZOL-loaded NPs and detected an increase in M1 macrophages (Qiao et al., 2021). Table 5

**TABLE 5:** The summary of available approaches of CM-coated NP in immunotherapy.

Purpose	Agent	Result
Remodeling TIME based on TAM	ZOL	Increasing M1-phenotype macrophages and decreasing M2-phenotype macrophages in the tumor microenvironment Qiao et al. (2021)
	Cu2-xSe NP	Reducing microenvironment hypoxia, increasing M1-phenotype macrophages, decreasing M2-phenotype macrophages in the tumor microenvironment Lee et al. (2022)
	DTX	Increasing M1-phenotype macrophages, and decreasing M2-phenotype macrophages in the tumor microenvironment Chen et al. (2021a)
Antigen presenting	Cancer cell membrane	Increasing CD8^+^ and CD4^+^ T-cells Chen et al. (2021a), activating NKs Wu et al. (2021)

TIME, tumor immunosuppressive microenvironment; TAM, tumor-associated macrophage; ZOL, zoledronate; DTX, Docetaxel; CD, cluster of differentiation; NK, natural killer cell.

CM-coated NPs also serve as artificial antigen presenters. Various outer membrane proteins and phospholipids in tumor CM-coated NPs can activate leukocytes and different immune responses (Zhang et al., 2019; Gong et al., 2020; Sushnitha et al., 2020).

As mentioned, homotypic CM-coated NPs pose therapeutic and drug delivery-assisting features. They can also activate immune responses against tumor cells while traveling to the tumor location in the body. (Jin et al., 2019) investigated this hypothesis on orthotopic glioma and breast cancer-bearing mice. They measured the immune response by quantifying and comparing CD4^+^ and CD8^+^ cytotoxic T-lymphocytes in the spleen and lymph nodes of orthotopic mice models and control group, which showed a significant increase in orthotopic mice. An increase in interferon-γ (IFN-γ) was also detected in the mentioned mice (Jin et al., 2019). Although the study did not test the GBM model, its strategy has inspired further immunotherapies in GBM. Chen et al. introduced a multifunctional CM-coated drug. The study coated PLGA NPs with DTX and Prussian blue NPs. PLGA NPs acted as chemotherapeutic agents. DTX remodels TIME and enhances M1-type, and reduces M2-type macrophages. The M2 macrophages decreased from 68.57% to 32.80%, and the M1 macrophages increased from 37.02% to 70.81%. Furthermore, NPs acted as efficient PTT transducers. The antigen-presenting in the study was accomplished. Results indicated an increment in DCs maturation rate and CTLs activation. CTLs also increased from 17.33% to 35.5% (Chen et al., 2021a).

The CM-coated NPs can enhance NKs activation. Wu et al. used magnetite (Fe_3_O_4_) coated with silicon dioxide (SiO_2_) and cancer CMs to activate NKs against GBM. Enzyme-linked immunosorbent assay was used for granzyme B, IFN-γ, and perforin measurement, which are released from activated NK. Flow cytometry was used to detect surface receptor expression on activated NK cells. The results were promising *in vitro,* but further studies are necessary to test the effect of tumor CM *in vivo* (Wu et al., 2021).

In a study using bacterial outer membrane vesicles of *Escherichia coli* as a source of the CM in producing CM-coated gold NPs, researchers examined the efficiency of this complex combined with radiotherapy or immunotherapy in GBM tumor-bearing mice. The result indicated that the gold nanoparticle coated with bacterial CM could effectively exert radiosensitizing and immune-modulatory effects, leading to tumor growth suppression in sub-cutaneous and *in-situ* GBM-bearing mice. This complex also resulted in a longer survival rate in *in-situ* tumor-bearing mice. Inducing ROS in glioma cells in response to the bacterial CM-coated gold nanoparticle carriers was responsible for the mechanism of this successful application (Chen et al., 2021b).

### 4.3 Gene therapy

Gene therapy was introduced in the 1970s and has brought much attention in the last 20 years. It refers to using genetic materials to treat diseases. There is a wide range of gene therapy approaches. Plenty of genes are usually responsible for cancers, so applying direct gene therapy is ineffective in cancer treatment. Hence, gene therapies in cancer usually are applied to enhance immune responses or kill cancer cells. In GBM, gene therapy approaches have succeeded in the pre-clinical experiment, and some clinical trials are ongoing. However, despite its clinical potential, the method has flaws, like a risky delivery system that must be improved in further investigations (Cross and Burmester, 2006; Kwiatkowska et al., 2013). In nucleic acids (NAs) delivery, the presence of carriers is necessary. Generally, there are two types of NAs carriers; viral and non-viral. Non-viral carriers are less likely to elicit immune responses. CM-coated NP is a non-viral gene delivery approach that can overcome many challenges in gene delivery, like NAs’ negative charge and relatively large size (Sung and Kim, 2019; Han et al., 2021; Scully et al., 2022).

As it was discussed, one of the gene therapy techniques in cancer treatment is killing cancer cells directly. This approach is called suicidal gene therapy. Herpes simplex virus thymidine kinase (HSV-TK) is a viral suicidal gene that has shown its efficacy in malignant brain tumor treatment. They cause an abnormality and stop cell division in the DNA replication phase (Hossain et al., 2020). In a successful animal model study, HSV-TK plasmid was covered with PEI and then coated with GBM-CM for more efficient non-viral gene delivery. The suicidal gene showed a better result when it was coated with CM than when it was naked or only covered with PEI. This study proves the delivery system’s importance in gene therapy and the efficacy of CM-coating for drug delivery (Han et al., 2021).

RNAi-based therapies have emerged as another therapeutic approach in gene therapy. This approach is successful in pre-clinical trials in cancer and GBM treatment. RNAi can regulate gene expression post-transcriptionally. They moderate the overexpression of dysregulated genes in different types of cancer. For example, in GBM, it has been used to repress genes that are responsible for vascularization and stem cell proliferation, like VEGF, epidermal growth factor receptor, and transcriptional signal transducer and activator (Guo et al., 2010; Lozada-Delgado et al., 2017; Mirzaei et al., 2021) Figure 5.

**FIGURE 5:**
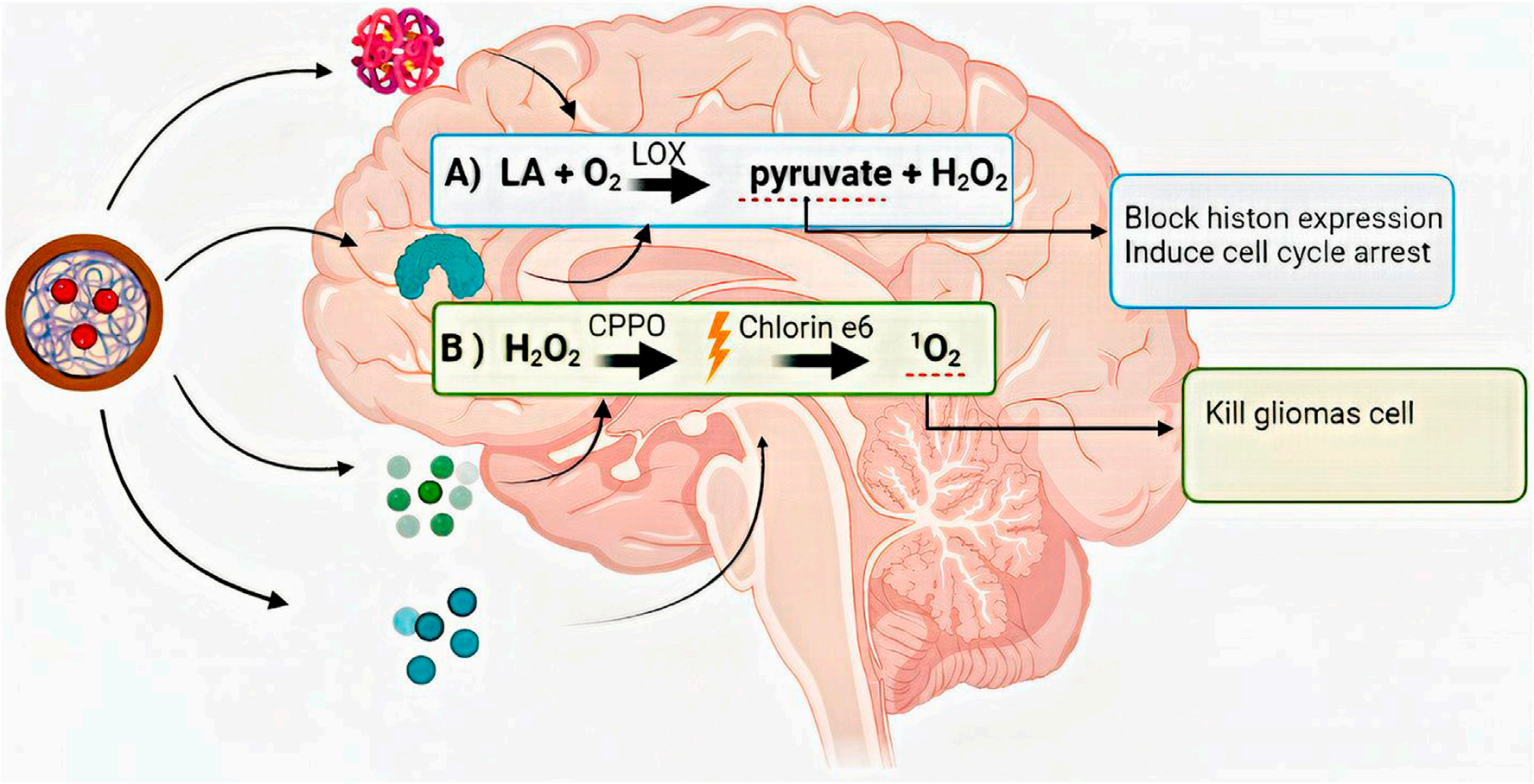
The summary of the anticancer reaction chain that was started and progressed with lactate oxidase, bisoxalate, and chlorine-6. LA: lactate, O_2_: Oxygen, H_2_O_2_: hydrogen peroxide, LOX: lactate oxidase, CPPO: bis [2,4,5-trichloro-6-(pentyloxy carbonyl)phenyl] oxalate, ^1^O_2_: cytotoxic singlet oxygen.

SiRNAs, micro RNAs, small hairpin RNAs, and long non-coding RNAs are different RNAi tested in GBM treatment. Like DNA, RNAis need a carrier to help it penetrate BBB and efficient cellular uptake (Mirzaei et al., 2021). CM-coating technology can introduce a new carrier for siRNA and other RNAis. For example, in one study, a three-layer multifunctional drug delivery system based on the RBC membrane, PEI, and citra conic anhydride grafted poly-L lysine (PLL-CA) was designed for siRNA delivery (Liu et al., 2020a). In another innovative study, the RBC membrane was modified with cyclo Arg-Gly-Asp-d-Phe-Cys (cRGD) peptide for better binding with cancer cells. The modified RBC could carry microRNA-137 to inhibit cell proliferation (Li et al., 2022a).

Gene therapy and CM-coating technology are both recent innovative studies that have shown attractive prospects for their combination. Nevertheless, more investigation is needed before starting the clinical phase.

### 4.4 Synergic phototherapy

Phototherapy as a therapeutic approach to cancer treatment was first introduced in 1970. Since then, there have been many advancements in this field. Phototherapy has several advantages compared to traditional anticancer treatments, such as fewer side effects and better penetration through the tight junctions of the BBB. Phototherapy is categorized into two groups: photodynamic therapy (PDT) and PTT, which will be further discussed (Fong et al., 2018; Shi and Sadler, 2020; Hak et al., 2021) (Table 6)

**TABLE 6:** A comparison between PDT and PTT.

Method name	Mechanism of method	Photosensitizers	Available synergic therapies with CM-coated NPs for GBM
PDT	Photosensitizers absorb light energy and pass it to oxygen to make specific cytotoxic molecules (ROS) that can destroy CMs, proteins, and cells Fong et al. (2018); Hak et al. (2021)	NPs like quantum dots, silica NPs, carbon nanomaterials, polymer NPs, and liposomes bind with photosensitizer drugs Abadi et al. (2021), chlorine-6 Lu et al. (2022)	Metabolism-based PDT synergic therapy Lu et al. (2022), PTT and PDT synergic therapy Liu et al. (2020d)
PTT	Laser radiation to photosensitizes can cause hyperthermia and destroy cancer cells Doughty et al. (2019); Hak et al. (2021)	NPs like gold, graphene oxide, and carbon nanotubes have been introduced as successful PTT agents Yang et al. (2012), ICG Liu et al. (2020d), GQDs Ren et al. (2022)	Chemotherapy and PTT synergic therapy Ren et al. (2022), PDT and PTT synergic therapy Liu et al. (2020d), gene and PTT synergic therapy Li et al. (2022a), Immunotherapy and PTT synergic therapy Chen et al. (2021a)

PDT, photodynamic therapy; PTT, photothermal therapy; CM, Cell membrane; ROS, reactive Oxygen species; NP, nanoparticle; ICG, indocyanine green; GQD, graphene quantum dot.

#### 4.4.1 PDT

PDT is based on a photosensitizer’s function. The photo-activation of photosensitizing molecules actively integrated into tumor tissue leads to the excitation of these molecules’ molecular oxygen to either the singlet or triplet state (Cramer and Chen, 2019). In the singlet state, the photosensitizer converts light energy to either heat or fluorescence. However, in the triplet state, ROS is produced, responsible for tumor cell destruction. In other words, photosensitizers can absorb light energy and pass it to oxygen. This process makes specific cytotoxic ROS that can destroy CMs, proteins, and membranes of intracellular organelles, leading to the activation of necrosis and apoptosis or triggering a subsequent immune response (Chernov et al., 2018; Fong et al., 2018; Hak et al., 2021). PDT in brain tumors exerts its anticancer effects by destroying tumor microvasculature (Abdurashitov et al., 2020). NPs with the modified surface are the third generation of photosensitizers that have not been approved by the FDA yet and demand more investigations (Yang et al., 2012; Cramer and Chen, 2020). PDT cannot be effective in advanced GBMs. Furthermore, it cannot cure GBM without a synergic approach (Fong et al., 2018; Abdurashitov et al., 2020). As discussed earlier, CM-coated NPs, as third-generation of photosensitizers, can accelerate combined therapies alongside targeted drug delivery and easy penetration through BBB.

**FIGURE 6:**
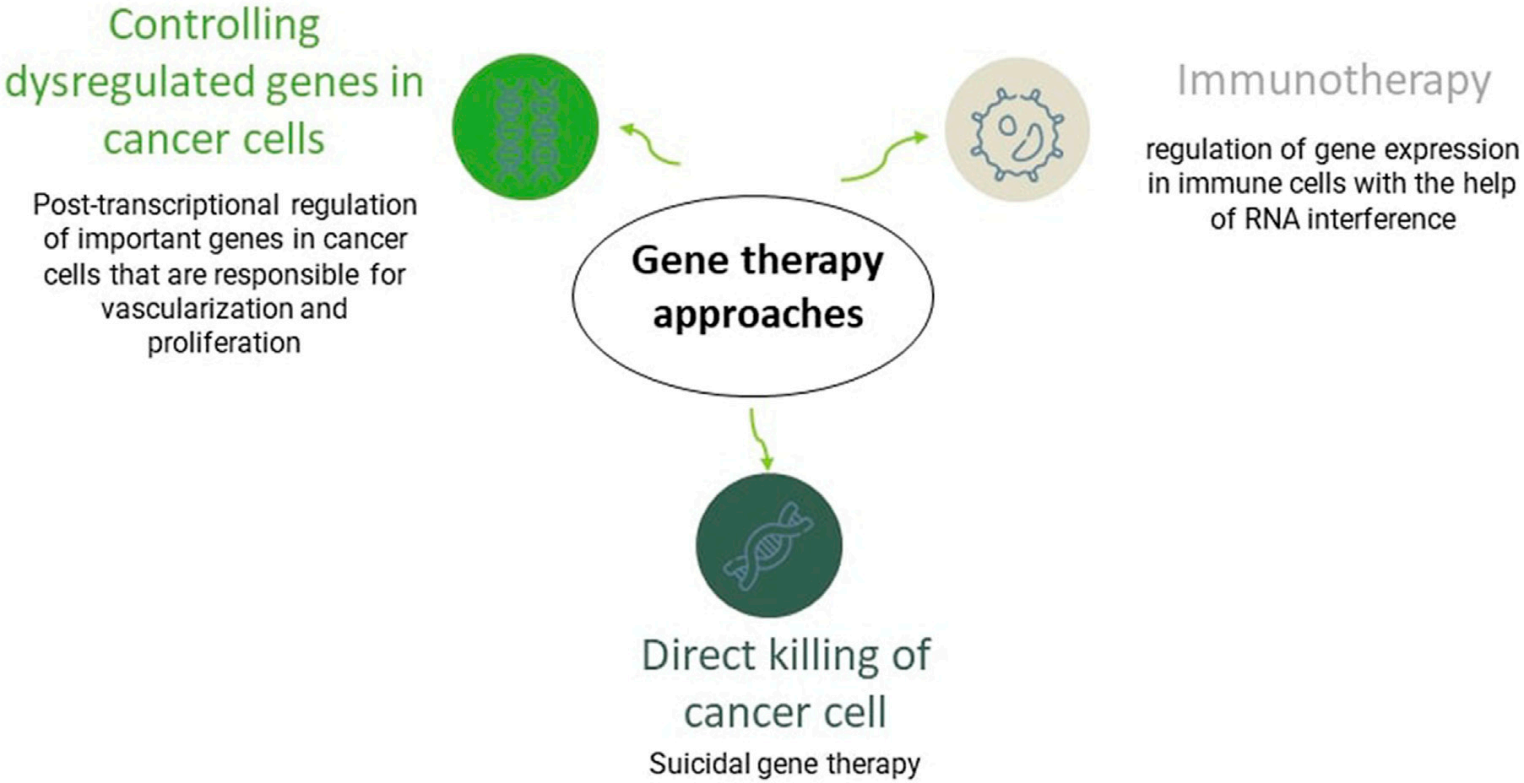
An overview of gene therapy approaches in GBM. Gene therapy can fight tumor cells via three main mechanisms, including the direct killing of them, termed as suicidal gene therapy, through enhancing the immune system response against the tumor, or via post-transcriptional modifying of essential genes responsible for tumor’s stem cell proliferation and vascularization like vascular endothelial growth factor gene.

For example, (Lu et al., 2022) devised a chain reaction system based on cell metabolism, PDT, and CM-coated NPs that had its anticancer effect in several steps. The summary of their anticancer reaction chain that was started and progressed with lactate oxidase, bisoxalate, and chlorine-6 is demonstrated in Figure 6 (Lu et al., 2022).

#### 4.4.2 PTT

PTT is another photo-based therapy for cancer. Despite PDT, the PTT effect is not dependent on oxygen and can apply in advanced GBM cases. PTT is considered a non-invasive treatment using an external near-infrared laser for radiantly destructing tumor cells and a photo-absorbing agent (PTA) to sensitize the tumor cells to radiation (Bastiancich et al., 2021). PTAs accumulate in the tumor site upon radiation, absorb the light energy, and convert it into heat (Hussein et al., 2018; Bastiancich et al., 2021). This process leads to hyperthermia and is responsible for fighting tumor cells and their ablation (Doughty et al., 2019; Hak et al., 2021).

In this regard, NPs possessing internal optical properties or those which can be incorporated with PTA agents are the best choices. For instance, gold, graphene oxide, and carbon nanotube NPs have been introduced as successful agents for the PTT of GBM (Yang et al., 2012). Alexa Guglielmelli et al. successfully designed keratin-coated gold NPs for PTT of GBM *in vitro* (Guglielmelli et al., 2020). Multiple studies were also conducted on CM-coated NPs and PTT in GBM for advanced drug delivery *in vivo*. An example of a successful study in this field is the co-delivery of CQDs and DOX, which was discussed before (Ren et al., 2022).

ICG is an FDA-approved photosensitizer for PTT. ICG can initiate both PTT and PDT reactions. Yu Peng Liu et al. designed micelle NPs containing ICG. It successfully restrained metastasis with the dual effect of PDT and PTT on neovascular endothelial cells and GBM cells (Liu et al., 2020d). ICG has been tested as an RBC membrane-coated NPs alongside microRNA and MSNs for synergetic gene and PTT (Li et al., 2022a). The ICG RBC membrane-coated NPs have also been successfully tested for tumor imaging (Wang et al., 2020).

## 5 Conclusion

It has not been long since CM-coating technology was introduced as a novel approach to drug delivery. Since then, numerous studies have examined this method using different techniques, cell lines, and NPs in various diseases, including cancers. Besides, a significant number of oncology studies in recent years suggest changing the conventional direction of cancer treatment, including surgery with radiotherapy and chemotherapy, to more advanced and novel therapy methods and medicines. Recently, CM-coated therapeutic agents were developed as novel treatment approaches for fighting poor prognostic diseases, including malignant and fatal cancers with low survival rates, like GBM. The efficacy of CM-coated NPs has been evaluated in different therapeutic approaches for GBM treatment, from chemotherapy to gene therapy and PTT. Some studies examined CM-coated NPs’ potential to penetrate through BBB and their antitumor activity only in a reconstructed laboratory model. On the other hand, some have examined them on animal models and the *in vitro* conditions. These *in vitro* and *in vivo* studies helped scientists evaluate the efficiency of CM-coated NPs as a single or combined therapy in GBM. The characteristics of CM vesicles provide an opportunity for simultaneously combined therapies. It allowed researchers to develop numerous new synergic treatments essential in cancers with high intratumoral heterogeneity like GBM. Scientists also compared the CM with materials like PEG that are used to cover NPs to escape immune invasion and for more prolonged circulation around the body. These studies demonstrated that CM-coated demonstrated much better results than bare or polymer-coated NPs.

The characteristics and application of CM-coated NPs, vary based on their membrane source and core NP. Various cell sources like red blood cells, cancer cells, MSCs, bacterial cells, and different leukocytes are appropriate for the membrane coating of NPs. The application of each CM varies Based on its unique characteristics. For example, some cell sources like cancer cells or leukocytes might serve as better candidates for immunotherapy. However, as the process of cancer membrane and erythrocyte membrane extraction is more convenient, most of the available studies in the field of CM-coated NPs for GBM utilized these two sources. Further investigations are required to assess the ability of other available membrane sources to penetrate BBB and target GBM cells. Alternatively, extensive NP with alternative characteristics and applications exist. An example is magnetic NPs, which are considered preferred candidates for synergic thermotherapies. More pre-clinical studies are required to find the best choice and optimized all-inclusive circumstances of CM-coated NPs. Moreover, there are still significant barriers on the road of CM-coated NPs to the clinic. Besides the high cost of these therapeutic approaches, their complex preparation process hinders mass production. In addition, their challenging storage condition may cause plenty of problems for patients and the clinic. Moreover, to date, there are no clinical trials evaluating the efficacy of CM-coated NPs and elucidating their long-term effects in humans. In conclusion, CM-coated NPs have shown promising effects in pre-clinical experiments for GBM treatment but have a long way to go to being widely used in humans and clinical trials. The promising results of applying CM-coated NPs in pre-clinical studies for GBM treatment may recommend future studies to investigate their effectiveness in treating other challenging CNS diseases in which BBB prohibits sufficient drug delivery, including Parkinson’s and Alzheimer’s diseases.

## Glossary

GBM: Glioblastoma multiform
GSC: GBM stem cell
BBB: blood-brain barrier
NP: nanoparticle
CM: cell membrane
CNS: central nervous system
WHO: world health organization’s
MRI: Magnetic resonance imaging
CD: cluster of differentiation
VEGF: Vascular endothelial growth factor
EPR: enhanced permeability retention
PEG: polyethylene glycol
NM: nanometer
FDA: food and drug administration
PTT: photothermal therapy
RNA: ribonucleic acid
RNAi: RNA interference
TMZ: Temozolomide
AMF: alternating magnetic field
ROS: reactive oxygen species
DOX: doxorubicin
SPIONs: superparamagnetic iron oxide nanoparticles
**MnO**_**2**_: manganese dioxide
PLGA: Polylactic-co-glycolic acid
DTX: docetaxel
PLA: polylactic acid
PEI: polyethylene imine
TEM: transmission electron microscopy
ICG: indocyanine green
RBC: red blood cell
WBC: white blood cell
DC: dendritic cell
NK: natural killer cell
TME: tumor microenvironment
TAM: tumor-associated macrophage
ZOL: zoledronate
MHC: major histocompatibility complex
TCR: T-cell receptor
CAR: chimeric antigen receptor
MSC: mesenchymal stem cells
CXCR: chemokine (C-X-C motif) receptor
CCR: C-C chemokine receptor
CDDP: cisplatin
H&E: hematoxylin and eosin
DOX: doxorubicin
BNNT: boron nitride nanotube
GQD: graphene quantum dot
TIME: tumor immunosuppressive microenvironment
IDO: indoximod
IFN-γ: interferon-γ
CTL: cytotoxic T lymphocyte
**SiO**_**2**_: silicon dioxide
BDEs: brain-derived exosomes
NA: nucleic acid
TAM: tumor-associated macrophage
TIME: tumor immunosuppressive microenvironment
HSV-TK: herpes simplex virus thymidine kinase
siRNA: Small interfering RNAs
PDT: photodynamic therapy
